# Exceptional treatment responses and molecular markers among 103 colorectal cancer cell lines

**DOI:** 10.1186/s43556-026-00551-z

**Published:** 2026-08-25

**Authors:** Anita Sveen, Jakob M. Stenersen, Theophilus Quachie Asenso, Solveig K. Klokkerud, Christian Kranjec, Ina A. Eilertsen, Bjarne Johannessen, Edward Leithe, Kushtrim Kryeziu, Manuela Zucknick, Ragnhild A. Lothe

**Affiliations:** 1 Department of Molecular Oncology, Institute for Cancer Research, Oslo University Hospital, P.O. Box 4953 Nydalen, Oslo, NO-0424 Norway; 2 Institute of Clinical Medicine, Faculty of Medicine, University of Oslo, Oslo, Norway; 3 Oslo Centre for Biostatistics and Epidemiology, Department of Biostatistics, University of Oslo, Oslo, Norway; 4 Oslo Centre for Biostatistics and Epidemiology, Oslo University Hospital, Oslo, Norway

**Keywords:** Colorectal cancer, Cell lines, Drug sensitivity testing, Exceptional treatment response, Prediction biomarkers, Transcriptomics

## Abstract

Patients with colorectal cancer have few molecularly guided treatment options. Cancers with exceptional treatment response are valuable models for discovery of molecular response mechanisms and might reveal new predictive biomarkers to guide treatment selection. However, the rarity of events is a clinical challenge. We performed integrated high-throughput drug sensitivity testing (*n* = 620 drugs) and molecular analyses of 103 colorectal cancer cell lines to build an in vitro foundation for exceptional treatment responses and their molecular markers. Exceptional responses were scored by a two-way outlier detection approach and were identified in 67 unique cell line-drug pairs (0.13% of all pairs tested). This involved 35 cell lines (34.0%) and 49 drugs (7.9%) representing standard, experimental, and non-oncology drugs of diverse classes. Plausible response mechanisms on the gene, protein, and/or pathway expression levels were identified at baseline in 89.6% of the 67 pairs. Experimental validation confirmed treatment-induced marker suppression in seven of eight selected pairs, including target engagement of kinase inhibitors and downregulation of markers of extended response mechanisms. Recurrent exceptional sensitivity to the same drug in different cell lines involved functionally convergent molecular mechanisms, but rarely precisely the same marker. Most exceptional sensitivities (77.8%) could be predicted with multivariable transcriptomic models. In conclusion, this study provides in vitro support for biomarker-guided prescreening to potentially obtain exceptional treatment response to diverse drugs and therapeutic targets in colorectal cancer. Most exceptional responses have a clear molecular underpinning. The study also provides a large and openly available pharmaco-transcriptomics resource for colorectal cancer cell lines.

**Supplementary Information:**

The online version contains supplementary material available at 10.1186/s43556-026-00551-z.

## Introduction

Cancers with exceptional response to systemic therapy are particularly attractive models for investigation of molecular response mechanisms [1]. Extreme treatment responses are expected to have a strong molecular underpinning and might reveal new predictive biomarkers to guide treatment selection also in patients with more conventional outcomes [2]. An illustrative example is inactivating mutations of two negative regulators of mTOR, *TSC1* and *NF2*, in a bladder cancer with complete and durable response to the mTOR inhibitor everolimus [3]. Notably, this case was reported from a clinical trial that failed to meet its primary endpoint with respect to treatment efficacy [4]. This illustrates the challenge that exceptional treatment responses are rare and often dismissed in clinical trials [5]. Most of the cases are anecdotal and reported in an N-of-1 setting [6].

Studies such as The National Cancer Institute Exceptional Responders Initiative have provided a more systematic evaluation of this rare subset of patients [7]. The initiative performed comprehensive molecular profiling of a collection of 111 exceptional responders and suggested a plausible response mechanism in 23% [8]. The mechanisms were strongly influenced by the type of treatment, and synthetic lethality between alterations of DNA repair pathways and cytotoxic chemotherapies was commonly observed. However, current knowledge of the molecular underpinnings of exceptional treatment response is limited to a few drugs per cancer type. For example, all three colorectal cancers (CRCs) in the Exceptional Responders Initiative were treated with chemotherapy [8]. CRC is the second most common cause of cancer-related deaths worldwide, and only few patients benefit from molecularly guided treatment [9, 10]. Most patients with advanced disease receive combination chemotherapy as first-line treatment, although immune checkpoint inhibitors have become the preferred choice for CRCs with microsatellite instability (MSI) and addition of dual BRAF-EGFR blockade to first-line chemotherapy can improve the outcome in *BRAF* p.V600E mutated cancers [11, 12]. Immune checkpoint inhibitors can have a profound treatment effect, and a high frequency of complete response to neoadjuvant treatment has been reported in early-stage MSI tumors [13, 14]. This supports the potential to achieve exceptional treatment response in CRC, and studies investigating a broader range of drugs can form the basis for new exceptional responses and expand our knowledge of the molecular mechanisms underpinning these outliers.

High-throughput drug sensitivity testing of cancer cell lines represents a valuable approach to uncover the molecular basis for treatment response [15]. However, pharmacogenomic interactions might depend on the tissue lineage [16], and cell lines of different lineages vary greatly in their fidelity to the original cancer type [17]. CRC is among the cancer types for which well-representative cell line models are available, and the molecular profiles of conventional CRC cell lines are similar to tumors [18, 19]. CRC cell lines can also model exceptional response to selected kinase inhibitors [20], but further discovery is dependent on larger datasets and testing of additional drugs.

In this study, we aimed to build an in vitro basis for exceptional treatment response in CRC and performed large-scale drug sensitivity testing (*n* = 620 drugs) and transcriptomics of 103 conventional CRC cell lines to detect outlier drug sensitivities and their molecular markers. We identified 67 exceptional sensitivities in 35 cell lines, with plausible molecular response mechanisms at baseline for the majority. Selected targets and markers were validated by reduced protein and/or phospho-protein levels after treatment. The study provides in vitro support for potential to obtain exceptional treatment response to diverse drug classes and drug targets in CRC, and strong molecular underpinnings support the potential for biomarker-guided approaches.

## Results

### In vitro pharmaco-transcriptomic compendium of CRC

**Fig. 1:**
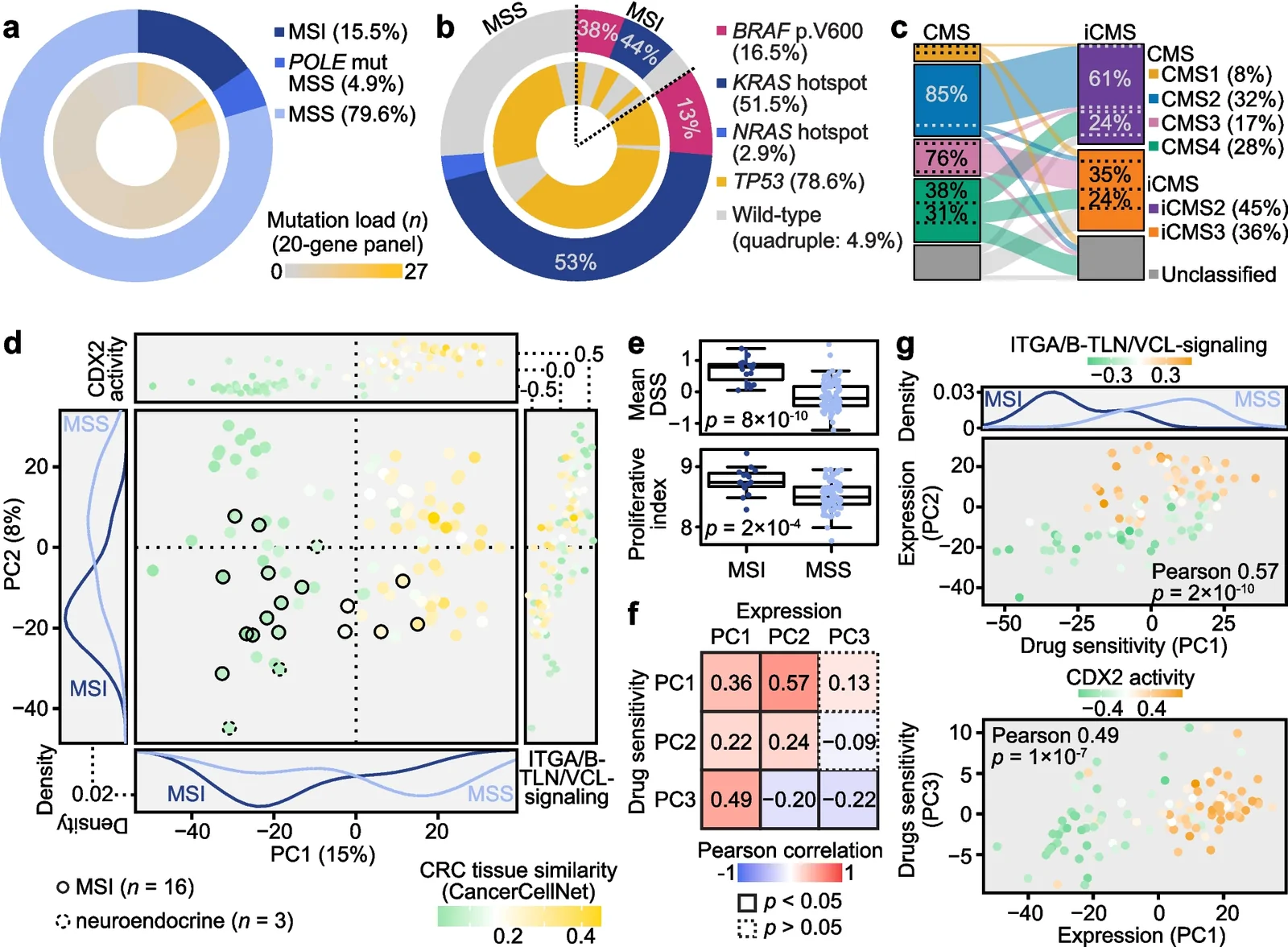
Overview of in vitro pharmaco-transcriptomic compendium. Circos plots of (**a**) MSI and *POLE* mutation status (outer circle) relative to the total mutation load (all nonsynonymous mutations in the 20-gene panel; inner circle), and (**b**) hotspot mutations of *BRAF* (p.V600) and *KRAS*/*NRAS* (p.G12, p.G13, p.Q61, p.K117, p.A146; outer circle) relative to *TP53* mutation status (inner circle) of MSI and MSS samples in the CRC cell line collection (*n* = 103). **c** Alluvial plot of CMS and iCMS classification concordance among cell lines. Sample overlaps between CMS and iCMS classes are indicated relative to the total number per subtype. **d** Transcriptomic PCA plot (middle) of all cell lines based on genes with highest expression variation (*n* = 3,000). Cell lines are colored according to the CancerCellNet CRC tissue similarity score. MSI and neuroendocrine cell lines are marked with black outlines, as indicated. Scatter plots on the top and right show correlation between PC1 or PC2 and single-sample expression enrichment scores of CDX2 activity or the ITGA/B-Talin/Vinculin signaling pathway, respectively (both from the KEGG Medicus collection). Density plots on the bottom and left show the distribution of PC1 and PC2, respectively, according to MSI status. **e** Boxplots of mean DSS values (scaled and centered; top) for a filtered set of drugs (*n* = 203) and a transcriptomic proliferative index (bottom) according to MSI status of the cell lines. *p*-values are from Student’s t-tests. **f** Heatmap of Pearson correlations of PC1-PC3 of all cell lines based on gene expression (*n* = 3,000 genes) versus drug sensitivity (*n* = 326 filtered drugs). Black and dashed outlines indicate the statistical significance level. **g** Scatter plots of the two most strongly correlated combinations of principal components on the gene expression versus drug sensitivity levels. Cell lines are colored according to single-sample enrichment scores of two signatures, as indicated. The density plot on top shows the distribution of PC1 on the drug sensitivity level according to MSI status of the cell lines

An in-house collection of conventional CRC cell lines (*n* = 103; Table S1) was analyzed by transcriptomic profiling and drug sensitivity screening (*n* = 461 or 528 drugs; Table S2). The cell lines have previously been sequenced for common CRC mutations (*n* = 20 genes) [21] and partly analyzed for protein expression on reverse-phase protein arrays (*n* = 297 proteins and phospho-proteins in 33 cell lines) [18]. The cell lines were selected to match the frequency of MSI among sporadic CRCs (15.5%) and were representative also of other main genomic and transcriptomic features of human tumors (Fig. 1a-c). Principal component analysis (PCA) on the transcriptomic level separated the cell lines primarily according to CRC tissue similarity (Fig. 1d), and PC1 correlated strongly with estimates of CRC tissue similarity according to the CancerCellNet approach (Pearson correlation 0.87, *p* < 2 × 10^–16^) [17]. Both PC1 and the CRC similarity score correlated strongest to the single-sample enrichment score of CDX2 transcriptional activity among the gene sets in the KEGG Medicus collection (*n* = 658 gene sets; Pearson correlations 0.79, *p* < 2 × 10^–16^), consistent with the critical role of CDX2 in controlling intestinal cell differentiation [22]. MSI cell lines clustered in PCA (distinct from microsatellite stable [MSS] cell lines along both PC1 and PC2; *p* = 0.002 and 1 × 10^–4^, respectively) and had lower CRC tissue similarity scores than MSS cell lines (*p* = 0.01; all by Student’s t-test), consistent with the lower intestinal differentiation grade of MSI tumors [23].

The cell lines recapitulated well-known pharmacogenomic associations, including sensitivity to the BRAF V600E inhibitor encorafenib in cell lines with the corresponding mutation, sensitivity to the MDM2-TP53 interaction inhibitor idasanutlin in *TP53* wild-type cell lines, as well as resistance to the EGFR inhibitor afatinib and sensitivity to the multi-kinase inhibitor regorafenib and the MAP2K1/2 inhibitor trametinib in cell lines with *RAS* hotspot (*KRAS*/*NRAS* p.G12, p.G13, p.Q61, p.K117, p.A146) or *BRAF* p.V600 mutations (Fig. S1a). PCA based on drug sensitivity scores (DSS) showed a major distinction between MSI and MSS cell lines along PC1 (*p* = 3 × 10^–11^ by Student’s t-test; Fig. S1b). The MSI cell lines were generally more drug sensitive and had a higher mean DSS across the drug panel (Fig. 1e and Fig. S1c). This corresponded with a higher proliferative index of MSI cell lines on the gene expression level (Fig. 1e). However, there was only a weak correlation between the proliferative index and mean DSS among cell lines in general (Pearson correlation 0.29, *p* = 0.003).

Comparisons of the transcriptomic and pharmacological profiles of the cell lines by correlation analyses of the 3 first principal components from PCA showed correspondence between the two data levels (Fig. 1f). Strongest correlation was found for PC1 of DSS values and PC2 of gene expression, as well as PC1 of gene expression and PC3 of DSS values (Fig. 1g). This supports the potential for transcriptomic modeling of drug sensitivity.

### Two-way outlier analysis defines in vitro exceptional drug sensitivities

Exceptional drug sensitivities were identified as outlier DSS values scored in both sample-wise and drug-wise analyses of the drug sensitivity matrix (*n* = 103 cell lines and 620 drugs). This approach identified 80 unique cell line-drug pairs, of which 13 were excluded due to low confidence in the dose–response curves (flat curves; Fig. S2). The remaining 67 pairs (0.13% of the 50,716 pairs tested) were scored as confident exceptional drug sensitivities (Fig. S3 and S4). Most of these were novel, while inclusion of three previously described outlier drug activities supported the validity of the approach. This included FGFR inhibitors in the cell line NCI-H716, an ALK inhibitor in C10, and a PORCN inhibitor in SNU-1411 (Fig. S5a-c) [20, 24]. Furthermore, the cell lines KM12 and SK-CO-1 had outlier sensitivity to TRK and PARP inhibitors, respectively, which has previously been proposed to be associated with a *TPM3*-*NTRK1* fusion and biallelic *ATM* mutation [25, 26]. However, the drugs did not have outlier activity in the respective cell lines and were therefore not scored as exceptional, illustrating the impact of analyzing a large drug library in the sample-wise outlier detection approach (Fig. S6).

**Fig. 2:**
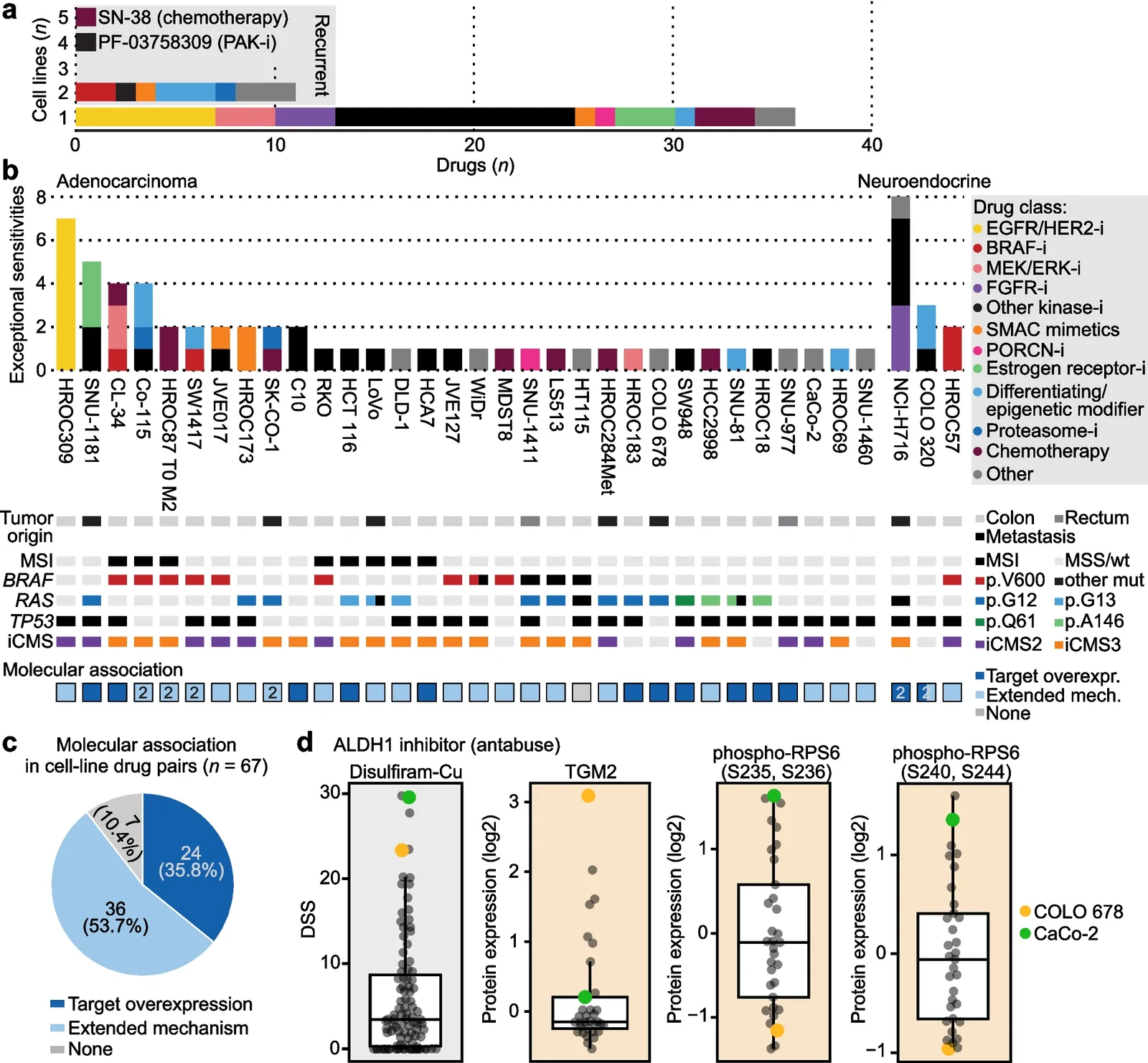
Summary of exceptional drug sensitivities. **a** Horizontal bar plot of drugs with exceptional activity (total *n* = 49) according to the number of cell lines with exceptional sensitivity. Drugs with exceptional activity in more than one cell line were considered recurrent, and the two drugs with most frequent exceptional activity are specified. Drug classes are colored as indicated in panel b. **b** Bar plot of the number of exceptional drug sensitivities per cell line. Cell lines (*n* = 35) are sorted by decreasing number of exceptional drug sensitivities, MSI status, mutation status, and iCMS classification as indicated in the panels below. Three neuroendocrine cell lines are plotted separately. The bottom panel indicates the category of molecular association to the exceptional drug sensitivity. Six cell lines with molecular associations to two distinct drugs are indicated. **c** Pie chart of the frequency of each category of molecular association among all cell line-drug pairs with exceptional sensitivity (*n* = 67). **d** Box plots of DSS values of disulfiram-copper (gray background) and (phospho-)protein expression values of a drug target or proposed marker (orange background) among cell lines (*n* = 99 and 33 cell lines for DSS and protein data, respectively). Two cell lines with exceptional sensitivity are highlighted. Protein expression values are median centered and phosphorylation sites are specified

The exceptional sensitivities were found in 35 cell lines (34.0%) and for 49 drugs (7.9%) representing a variety of drug classes (Fig. S7 and Table S2). All drugs except two had exceptional activity in only one or two cell lines each (Fig. 2a). The exceptions included SN-38, which is the active metabolite of the chemotherapy irinotecan, with exceptional activity in five cell lines (MSS: HROC284Met, LS513, MDST8, SK-CO-1; and MSI: HROC87 T0 M2), and the PAK4 inhibitor PF-03758309 in four cell lines (MSI: Co-115, HCT 116, RKO; and neuroendocrine: COLO 320). Similarly, most of the cell lines (62.9%) had exceptional sensitivity to a single drug each, and cases of multiple exceptional sensitivities in the same cell line commonly converged on drugs with the same or similar mechanism of action (Fig. 2b). The most prominent example was exceptional sensitivity to seven different EGFR/HER2 inhibitors in HROC309, which is an MSS and *RAS*/*BRAF* wild-type cell line derived from a left-sided colonic tumor. All three neuroendocrine cell lines had more than one exceptional sensitivity, consistent with their distinct histology.

Validation analyses in the publicly available Genomics of Drug Sensitivity in Cancer (GDSC2) dataset [15] showed strong correlations of drug sensitivities for overlapping cell lines (*n* = 34; median Pearson correlation −0.81, 95% confidence interval −0.84 to −0.78) and drugs (*n* = 63 analyzed; median Pearson correlation −0.65, 95% confidence interval −0.69 to −0.61; Fig. S8a), although the GDSC2 and in-house datasets were based on different computational response metrics (natural logarithm of the fitted IC50 value and DSS, respectively). All drugs with weak correlations (|Pearson|< 0.4, *n* = 10) were screened with a tenfold lower concentration range in the GDSC2 dataset than the in-house experiments (1,000-fold versus 10,000-fold). All overlapping cell line-drug pairs with exceptional sensitivity were highly correspondent between the two datasets, supporting technical reproducibility for identification of exceptional drug sensitivities (Fig. S8b).

Adenocarcinoma cell lines with at least one exceptional sensitivity were more frequently of the transcriptomic intrinsic consensus molecular subtype iCMS3 than iCMS2 (odds ratio [OR] 3.0, *p* = 0.02 by Fisher’s exact test) and had lower CRC tissue similarity scores than cell lines with no exceptional sensitivity (*p* = 0.08 by Welch’s t-test). There was also enrichment with *BRAF* p.V600 mutations among the exceptional responders (OR 3.0, *p* = 0.02), but there was no significant difference in the frequency of MSI (OR 2.2, *p* = 0.16), *RAS* hotspots mutations (OR 0.5, *p* = 0.1), or *TP53* mutations (OR 0.5, *p* = 0.2; all by Fisher’s exact test). The exceptional responders had stronger drug sensitivities in general, estimated as a higher mean DSS across the drug panel (*p* = 0.02 by Welch’s t-test).

### Most exceptional drug sensitivities have a clear molecular underpinning

Further molecular associations of the exceptional drug sensitivities were investigated on the gene, protein, and pathway expression levels (the latter estimated as single-sample enrichment scores of a custom gene set collection; Table S3) and guided by the mechanism of action of each drug. Plausible response mechanisms were detected in 60 (89.6%) of the 67 cell line-drug pairs, and all cell lines except one (*n* = 34 of 35, 97.1%) had a proposed mechanism for at least one exceptional sensitivity (Fig. 2b-c and Table 1). These included the three previously described associations between *FGFR2* overexpression and FGFR inhibitor sensitivity in NCI-H716 (*FGFR2* amplification), *ALK* overexpression and ALK inhibitor sensitivity in C10 (*EML4*-*ALK* fusion), and *RSPO3* overexpression and PORCN inhibitor sensitivity in SNU-1411 (*PTPRK*-*RSPO3* fusion; Fig. S5a-c) [20, 24]. The neuroendocrine cell line NCI-H716 additionally had exceptional sensitivity to four other kinase inhibitors, associated with overexpression also of *AKT1* and *RET* (Fig. S5d).

**Table 1:** List of CRC cell lines with exceptional drug sensitivity and the proposed molecular underpinnings

Cell line	Drugs with exceptional activity^a^	Category of molecular association^b^	Molecular markers		
			High gene expression	High protein expression^c^	High pathway enrichment score^d^
HROC309	Afatinib; canertinib; dacomitinib; erlotinib; gefitinib; neratinib; tesevatinib	Extended mechanism	*ARHGAP8*	na	Ca2 +/CaM via RYR; Ca2 +/CaM via CaMK
SNU-1181	Clomifene; tamoxifen; toremifene	Target overexpression	na	na	Estrogen response (early)
	Radotinib; tivozanib	na	na	na	na
CL-34	Encorafenib; PD0325901; SCH772984	Target overexpression	*MAP2K2*	MAPK14 (pT180, pY182)	na
	8-amino-adenosine	na	na	na	na
Co-115	Lonafarnib; tipifarnib	Extended mechanism	na	AKT (pS473); AKT (pT308)	na
	PF-03758309		na	AKT (pS473); AKT (pT308); XIAP	na
	Oprozomib	na	na	na	na
HROC87 T0 M2	Eribulin	Extended mechanism	na	na	MYC targets; E2F targets; translation
	SN-38	Extended mechanism	*BCL2*	na	E2F targets
SW1417	Dabrafenib	Extended mechanism	*AURKA*	na	AURKB targets
	BAY 87–2243				
JVE017	LCL161	Extended mechanism	*BCL2L11*	na	IFN-RIPK1/3 signaling; hypoxia via HIF1A
	OTS-964	na	na	na	na
HROC173	Birinapant; LCL161	Extended mechanism	na	na	Apoptosis (v1); EGF-JAK-STAT signaling; IFN-γ response
SK-CO-1	SN-38	Extended mechanism	*BCL2L2*	na	na
	Oprozomib	Extended mechanism	na	na	Inflammatory response (TGFB1); PKC pathway
C10	CEP-37440; gilteritinib	Target overexpression	*ALK*	na	ALK signaling in cancer; PKC pathway
RKO	PF-03758309	Extended mechanism	*BCL2A1*	MCL1; CD274	na
HCT 116	PF-03758309	Target overexpression	*PAK5*	na	na
LoVo	7-hydroxystaurosporine	Extended mechanism	na	CAV1	TRKA receptor activation
DLD-1	Daporinad	Extended mechanism	na	na	NAD metabolism; reactive oxygen species pathway
HCA7	Triciribine	Target overexpression	*AKT3*	na	na
JVE127	PF-00477736	Extended mechanism	*PDK1*	na	mitosis (LIN9 targets)
WiDr	NMS-873	Extended mechanism	na	VHL	na
MDST8	SN-38	Extended mechanism	*BCL2A1*	na	na
SNU-1411	LGK974	Extended mechanism	*RSPO3*	na	na
LS513	SN-38	Extended mechanism	na	na	BCL2 overexpression and intrinsic apoptosis; metabolic reprogramming in CRC
HT115	NMS-873	na	na	na	na
HROC284Met	SN-38	Extended mechanism	*BCL2L1*; *BCL2L15*	na	CyclinB:CDK1 complex inactivation (CHEK1/2-mediated); mitosis (LIN9 targets); E2F targets
HROC183	RO5126766	Target overexpression	*MAP2K6*	na	KRAS mut signature; BRAF mut-like
COLO 678	Disulfiram-copper	Target overexpression	na	TGM2	na
SW948	AMG-900	Target overexpression	*AURKB*	AURKB; RB1; RB1 (pS807, pS811)	AURKB targets
HCC2998	Floxuridine	Extended mechanism	na	MUC1	na
SNU-81	ARV-825	Target overexpression	*BRD2*	na	na
HROC18	Triciribine	Target overexpression	na	na	PI3K-AKT-MTOR signaling
SNU-977	Sabutoclax	Target overexpression	*BCL2L11*; *BCL2L13*	na	BCL2 targets
Caco-2	Disulfiram-copper	Extended mechanism	na	RPS6 (pS235, pS236); RPS6 (pS240, pS244)	na
HROC69	ARV-825	Extended mechanism	na	na	H3K27Me3 high
SNU-1460	Salinomycin	Extended mechanism	*STAT3*	na	STAT3 targets down; IFNG pathway; IFN-RIPK1/3 signaling
NCI-H716	AZD4547; erdafitinib; LY-2874455	Target overexpression	*FGFR2*	na	FGFR2 signaling
	CCT196969; FRAX486; danusertib; ponatinib	Target overexpression	*FGFR2*; *RET; AKT1*	na	FGFR2 signaling; RAC-CDC42-PAK-ERK signaling
	Salinomycin	na	na	na	na
COLO 320	PF-03758309	Target overexpression	*PAK3; PAK5; BCL2*	na	na
	Lonafarnib; tipifarnib	Extended mechanism	*AKT3*	na	na
HROC57	Dabrafenib; encorafenib	Extended mechanism	*MAP2K6*	na	na

*na* not applicable

^a^Drugs with exceptional activity in the same cell line and with the same or similar mechanisms of action are listed in the same entry

^b^Two main categories (target overexpression and extended response mechanism)

^c^Protein phosphorylation sites (p) are indicated

^d^Pathway references are provided in Table S3

**Fig. 3:**
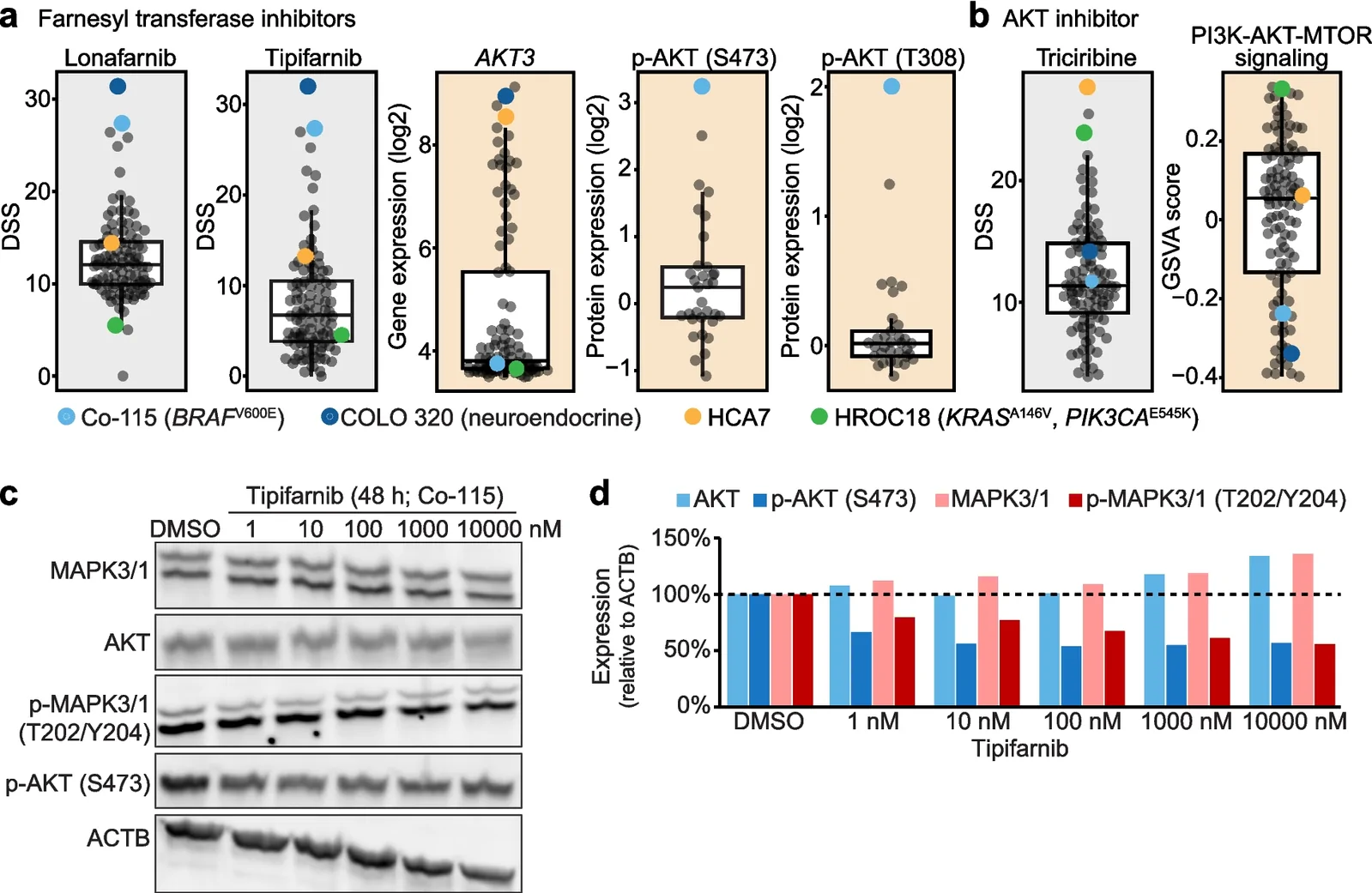
Examples of recurrent exceptional drug sensitivities and molecular markers. Box plots of DSS values of (**a**) two farnesyl transferase inhibitors and (**b**) the AKT inhibitor triciribine (gray background) and gene expression values or phospho (p)-protein levels of AKTs or single-sample enrichment scores (GSVA scores) of a pathway related to AKT activity (orange background) among cell lines (*n* = 100 or 101, 103, and 33 cell lines for DSS and gene and protein expression data, respectively). Four cell lines with exceptional sensitivity are highlighted in colors as indicated. Phospho-protein levels are median centered and phosphorylation sites are specified. **c** Western blots of Co-115 cells after treatment with DMSO (control) or five different concentrations of tipifarnib. All samples originate from the same experiment. The gel/blot with AKT and MAPK3/1 was processed in parallel to phospho-AKT and phospho-MAPK3/1. ACTB was used as loading control on both blots and is illustrated for the MAPK3/1 and AKT blot. Blots were run once per sample and experimental condition. **d** Quantification of Western blots in panel c relative to corresponding ACTB levels from each blot and centered based on expression after DMSO treatment

**Fig. 4:**
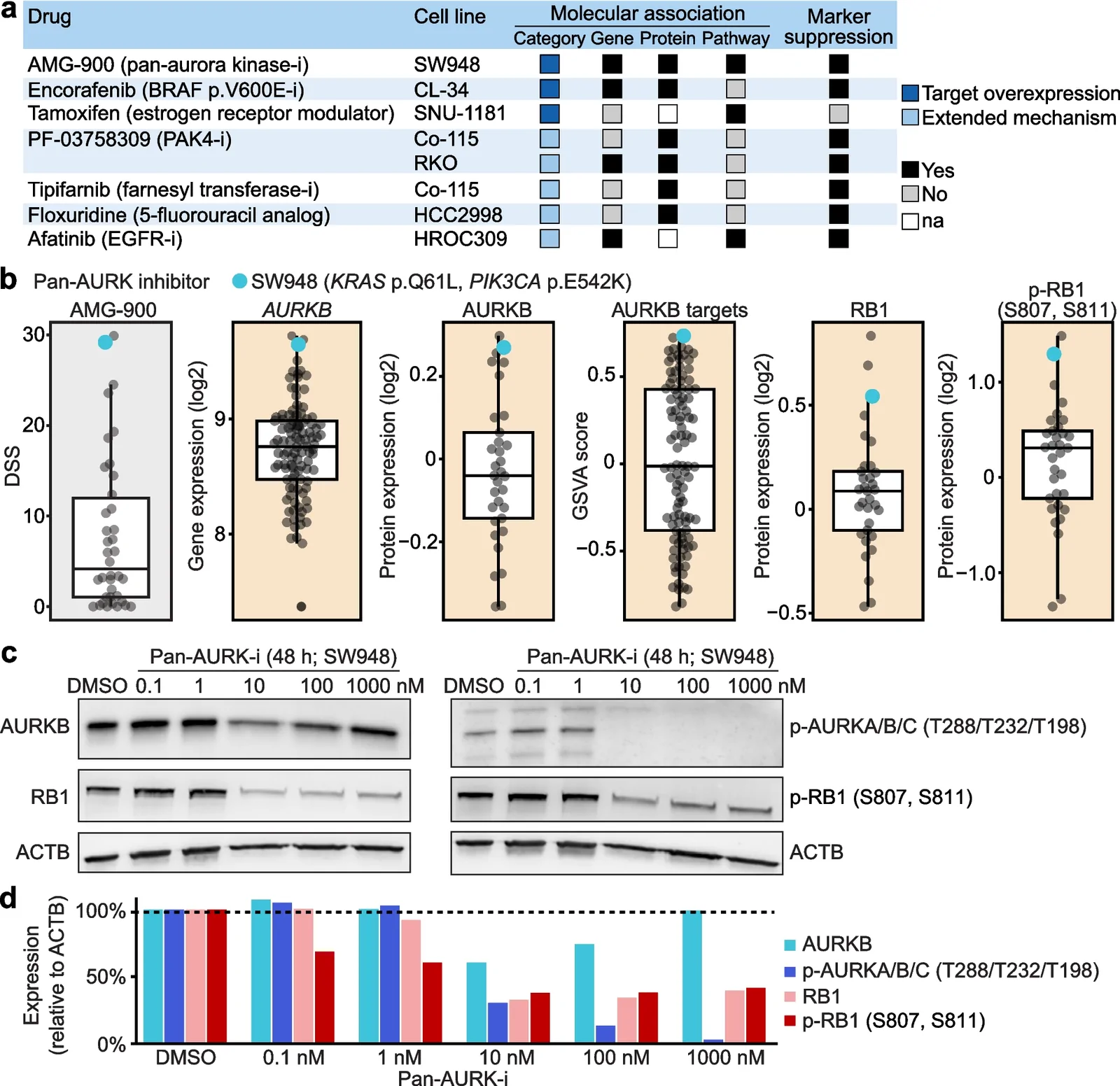
Drug target suppression in response to exceptional drug sensitivity. **a** Overview of post-treatment validation experiments of cell line-drug pairs with exceptional sensitivity (*n* = 8), including the category and data level of the proposed molecular associations in parental cell lines and results from evaluation of the proposed markers after treatment. **b** Box plots of DSS values of the pan-aurora kinase inhibitor AMG-900 (gray background) and gene or (phospho[p]-)protein expression values or single-sample enrichment scores (GSVA scores) of gene sets of drug target activity or other proposed markers (orange background) among cell lines (*n* = 38, 103 and 33 cell lines for DSS and gene and protein expression data, respectively). The cell line with exceptional sensitivity is highlighted in cyan. Protein expression values are median centered and phosphorylation sites are specified. **c** Western blots of SW948 cells after treatment with DMSO (control) or five different concentrations of AMG-900. Samples were derived from the same experiment and the gel/blot with AURKB and RB1 was processed in parallel to phospho-AURKA/B/C and phospho-RB1. ACTB was used as loading control on both blots. Blots were run once per sample and experimental condition. **d** Quantification of Western blots in panel c relative to corresponding ACTB levels from each blot and centered based on expression after DMSO treatment

**Fig. 5:**
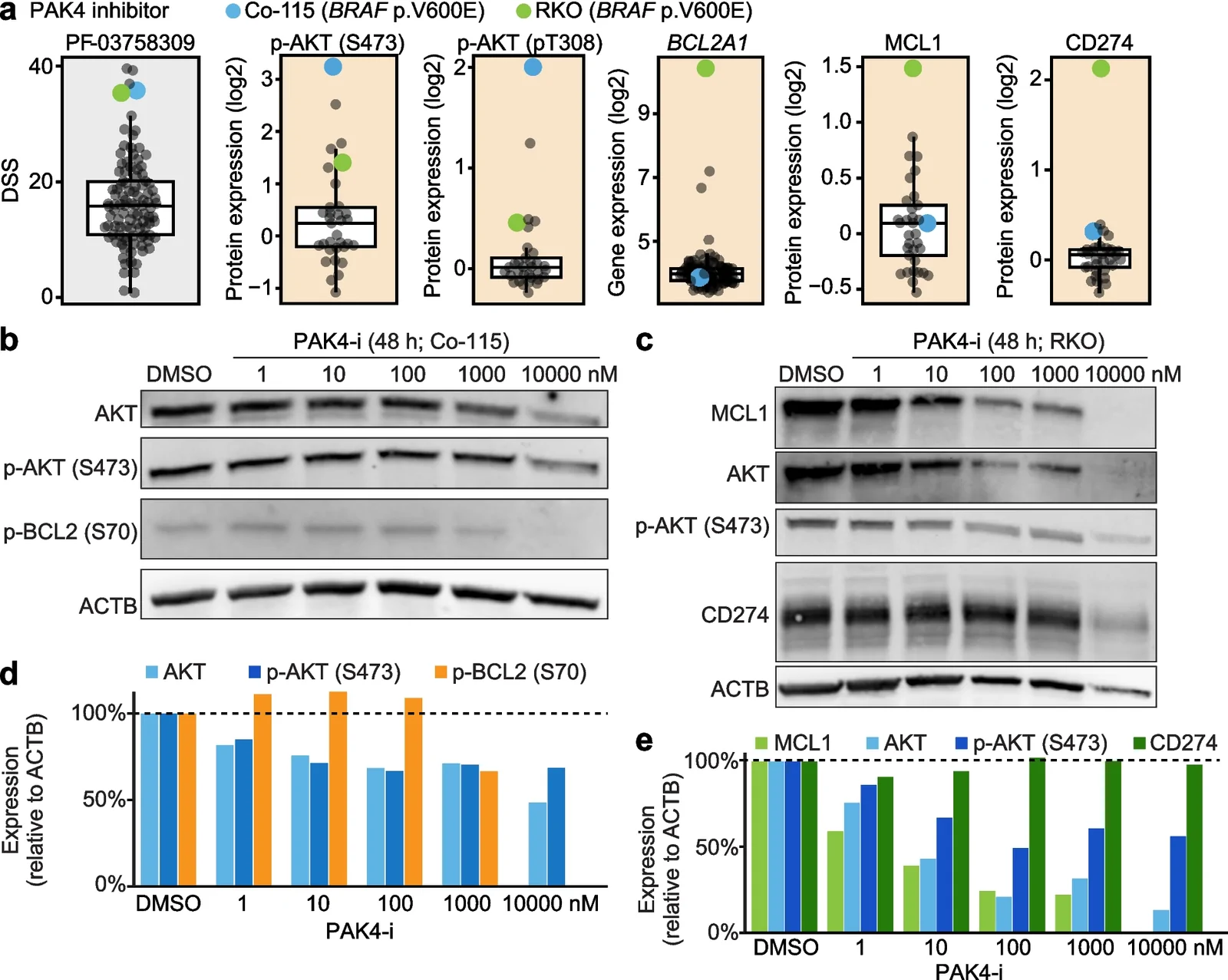
Engagement of extended response mechanisms in exceptional treatment response. **a** Box plot of DSS values of the PAK4 inhibitor PF-03758309 (gray background) and gene or (phospho[p]-)protein expression of selected markers (orange background) among cell lines (*n* = 103, 103 and 33 cell lines for DSS and gene and protein expression data, respectively). Two cell lines with exceptional sensitivity are highlighted in blue and green. Western blots of (**b**) Co-115 and (**c**) RKO cells after treatment with DMSO (control) or five different concentrations of PF-03758309. Samples originate from a single experiment per cell line. In panel b, the gel/blot with phospho-BCL2 was processed in parallel to AKT and phospho-AKT, and in panel c, the gel/blot with CD274 was processed in parallel to MCL1, AKT and phospho-AKT. ACTB was used as loading control on all blots and illustrated for the AKT/phospho-AKT blots. Blots were run once per sample and experimental condition. Quantification of western blots (**d**) in panel b and (**e**) in panel c relative to corresponding ACTB levels from each blot and centered based on expression after DMSO treatment

One of the exceptional responses not previously described is illustrated as an example in Fig. 2d, showing sensitivity to the alcohol-aversion drug disulfiram-copper in two cell lines with high expression of either the target protein TGM2 or phosphorylated RPS6, the latter potentially associated with ribosome biogenesis stress evoked by disulfiram [27]. All exceptional drug sensitivities (*n* = 35 cell lines and 67 cell line-drug pairs) and their molecular associations are summarized in Table 1 and illustrated in Figs. 2, 3, 4, and 5, Fig. S5, and Fig. S9 to S18. The proposed response mechanisms were based on overexpression of target genes/proteins/pathways in 13 cell lines (37.1%) and on extended molecular associations in 22 cell lines (62.9%; six cell lines had two proposed mechanisms; Fig. 2b). All the extended associations were considered plausible response mechanisms based on previous literature (Table S4).

### Recurrent events have similar molecular mechanisms

Recurrent exceptional sensitivity to the same (or similar) drug in more than one cell line (26.5% of drugs; Fig. 2a) was commonly associated with related molecular mechanisms that clearly converged on the same pathway and/or function but rarely involved precisely the same gene or protein. For example, recurrent exceptional sensitivity to the chemotherapy SN-38 was associated with high gene expression or pathway scores of different members of the BCL2 family of apoptotic regulators in all five cell lines (Fig. S9). Furthermore, exceptional sensitivity to farnesyl transferase inhibitors or an AKT inhibitor in two cell lines each was associated with high expression of *AKT3*, phosphorylated AKT, or the PI3K-AKT-mTOR pathway (Fig. 3a-b). Three of four cell lines with exceptional sensitivity to inhibitors of the Raf-MEK-ERK pathway had either *BRAF* p.V600E or *KRAS* p.G12D mutations in addition to high expression of *MAP2K2*, *MAP2K6*, or phosphorylated MAPK14 (Fig. S10). Exceptional sensitivity to targeting bromodomain and extraterminal (BET) family proteins was associated with high expression of the *BRD2* target gene in one cell line and with high pathway score of a histone methylation mark in another (Fig. S11a), consistent with the role of BET proteins in regulating transcription enhancer activity [28]. Two SMAC mimetics (LCL161 and birinapant) had exceptional activity in two cell lines with high expression of genes or pathways related to apoptosis, interferon and RIPK signaling, or hypoxia (Fig. S11b), consistent with the role of SMAC mimetics in regulating several cell death pathways [29].

In contrast, recurrent exceptional sensitivity to disulfiram-copper (described above and in Fig. 2d) and the PAK4 inhibitor PF-03758309 had diverse molecular associations. Sensitivity to PAK4 inhibition in four cell lines was associated with high expression of the *PAK3* and *PAK5* target genes, apoptotic markers, phosphorylated AKT, or immune checkpoint proteins (Fig. S12a).

Notably, overexpression of BCL2 family members was a common molecular association of exceptional sensitivity to four drugs with diverse mechanisms of action, including the BCL2 inhibitor sabutoclax (Fig. S12b) and three of the drugs described above (SN-38, a SMAC mimetic, and the PAK4 inhibitor). These data indicate that apoptotic markers are recurrently involved in sensitizing cell lines to strong therapeutic vulnerabilities.

### Drug targets are suppressed in response to exceptional drug activity

Eight cell line-drug pairs with exceptional sensitivities were selected for validation analyses of the proposed molecular underpinnings (Fig. 4a). The pairs were selected to cover drugs with various mechanisms of action and examples of both direct target engagement and extended molecular response mechanisms. One cell line with two different exceptional sensitivities (Co-115) and one drug with exceptional activity in two cell lines (the PAK4 inhibitor PF-03758309) were included. Validation experiments were performed by drug exposure at the same five concentrations used in the initial screen (Table S5), although with shorter incubation time (48 h versus 72 h). Strong effects on cell viability were confirmed with the highest drug concentrations (Fig. S19). Furthermore, suppression of the proposed molecular marker in response to treatment (relative to control treatment with DMSO) was evaluated by Western blotting and confirmed in 7 of the 8 selected cell line-drug pairs (Fig. 4a).

Exceptional sensitivity to the pan-aurora kinase inhibitor AMG-900 in the cell line SW948 (*KRAS* p.Q61L and *PIK3CA* p.E542K mutated) was associated with high gene and protein expression of AURKB, as well as a high pathway score for AURKB targets in parental cells (Fig. 4b). Western blotting confirmed target engagement by downregulated AURKB expression and decreased levels of phosphorylated AURKA/B/C after treatment with AMG-900 at the intermediate concentration (10 nM), and strongly diminished levels of phosphorylated AURKA/B/C at the highest concentrations (Fig. 4c-d). This concentration-dependent effect of AMG-900 corresponded with the dose–response curve of cell viability in the initial drug screen (Fig. S4).

Similarly, exceptional sensitivity to the BRAF V600E inhibitor encorafenib in the cell line CL-34 (*BRAF* p.V600E mutated) was associated with high gene expression of the downstream effector *MAP2K2* and a high phosphorylation level of MAPK14 in parental cells (Fig. S10a). Western blotting showed reduced levels of phosphorylated MAPK3/1 after treatment, confirming suppression of the MAPK pathway (Fig. S10b-c), although to only 50.7% of the original phosphorylation level even at the highest drug concentration. This was concordant with the incomplete cytotoxic effect of encorafenib in the initial drug screen (Fig. S3).

In contrast to these validation experiments confirming target suppression, the high pathway score for estrogen response found in association with exceptional sensitivity to estrogen receptor modulators in the cell line SNU-1181 (Fig. S13) could not be validated, since ESR1 was not detected by Western blotting in neither treated nor control samples of this cell line.

### Extended molecular mechanisms are engaged in exceptional treatment response

Two *BRAF* p.V600E mutated cell lines (Co-115 and RKO) had exceptional sensitivity to the PAK4 inhibitor PF-03758309. This was associated with a high level of phosphorylated AKT particularly in Co-115 (Fig. 5a), supported by the role of AKT in mediating the oncogenicity of PAK isoforms [30]. Both the total amount of AKT and the level of phosphorylated AKT were reduced in response to PAK4 inhibition in both cell lines (Fig. 5b-e). The parental RKO cell line also had outlier expression of the anti-apoptotic BCL2 family member MCL1, and PAK4 inhibition resulted in a concentration-dependent depletion of MCL1 (Fig. 5c and e), consistent with previous preclinical studies of T cell malignancies [31]. Furthermore, reduced phosphorylated AKT in Co-115 cells was confirmed as a response mechanism also for exceptional sensitivity to the farnesyl transferase inhibitor tipifarnib (Fig. 3c-d), consistent with previous mechanistic studies [32].

Exceptional sensitivity to floxuridine, an analogue of the standard chemotherapy 5-fluorouracil, was associated with outlier expression of MUC1 in the cell line HCC2998 (Fig. S14). MUC1 is described as a marker of chemoresistance [33], but was nonetheless strongly downregulated in response to treatment with floxuridine. The oncogenic C-terminal unit MUC1-C was not similarly affected.

Finally, treatment with the EGFR inhibitor afatinib in the exceptional responder cell line HROC309 resulted in reduced levels of total EGFR and phosphorylated EGFR (Fig. S15). This might be associated with outlier gene expression of *ARHGAP8* in the parental cells, since ARHGAP8 is a rho GTPase activating protein that can stimulate EGFR endocytosis and MAPK3/1 signaling [34], although we did not evaluate this mechanistic link in HROC309 cells.

As part of the validation experiments, SW948 cells treated with the pan-aurora kinase inhibitor AMG-900 were also evaluated for RB1 and phosphorylated RB1, based on high expression in the parental cell line (Fig. 4b). This supported reduced levels of both at intermediate drug concentrations (Fig. 4c-d), which is of interest considering that loss of *RB1* can lead to hyperdependency on AURKB for cell survival and to synthetic lethality with AURKA inhibitors [35, 36]. Furthermore, parental RKO cells also had outlier expression of the immune checkpoint ligand CD274 (PD-L1; Fig. 5a), which is of interest considering that PAK inhibition can stimulate anti-tumor immunity and improve the efficacy of immune checkpoint inhibition by downregulation of CD274 [37, 38]. However, there was no effect of PAK4 inhibition on CD274 expression in RKO in these validation experiments (Fig. 5c and e), which was likely due to the lack of an immune microenvironment in the cell cultures.

### Most exceptional drug sensitivities are predicted with multivariable transcriptomic models

**Fig. 6:**
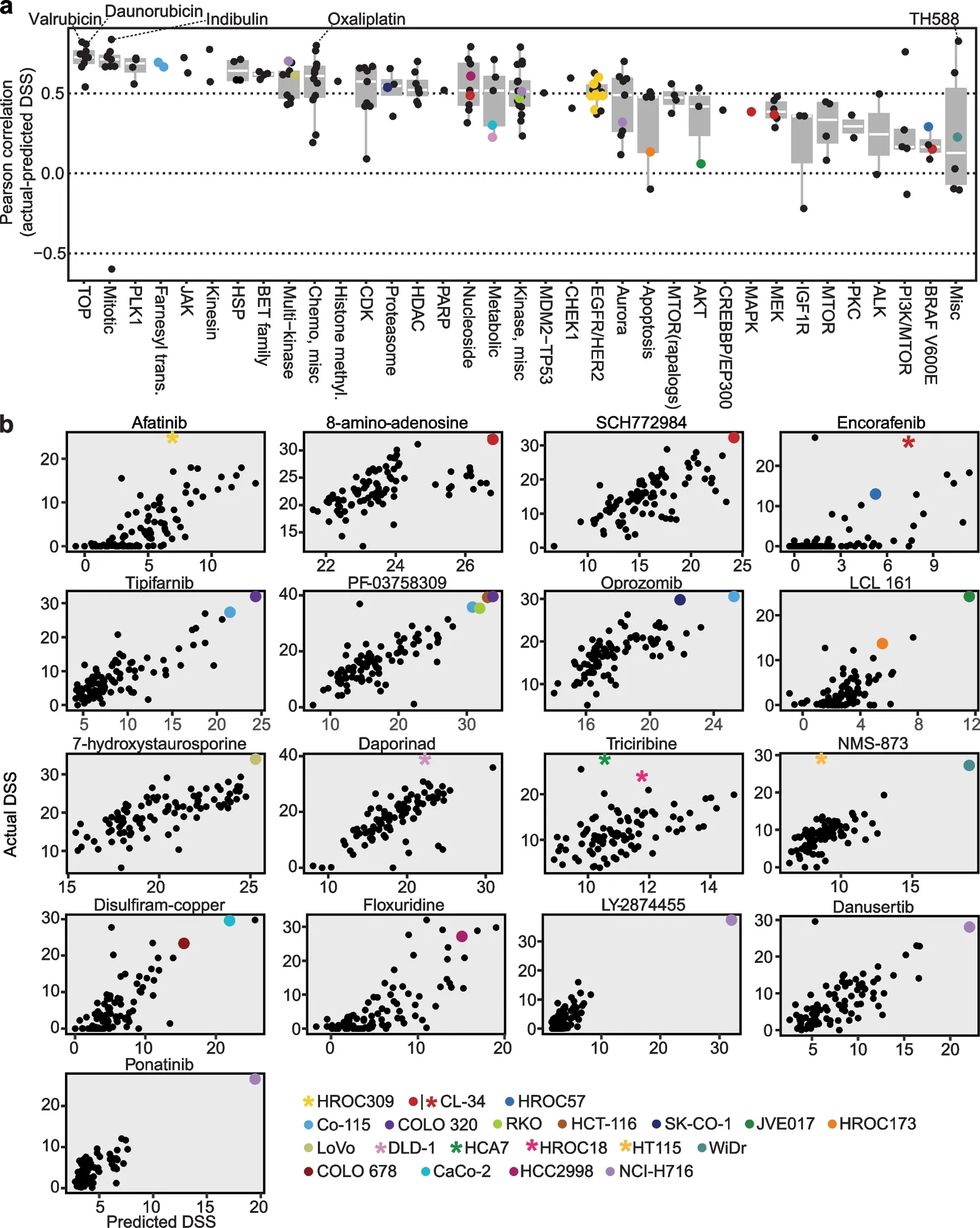
Transcriptomic prediction of exceptional drug sensitivity. **a** Box plot of Pearson correlations between actual and predicted DSS of each drug (*n* = 183; no model/genes for 20 drugs), estimated in the test set of cell lines. Results are from MADMMplasso models with MSI status as the interaction term. Drugs are grouped according to molecular target (Table S6) and ordered according to decreasing median correlation. The top 5 drugs are specified. Modeled drugs with exceptional activity are colored in correspondence with the cell-line drug pairs specified in panel b or in Fig. S21a. **b** Scatter plots of actual (vertical axes) and predicted (horizontal axes) DSS of drugs with exceptional activity. Cell lines with exceptional sensitivity are indicated by colors, and asterisks indicate exceptional sensitivities that were not well predicted

The regularized multi-response model MADMMplasso was used to build gene expression-based prediction models of drug sensitivity [39]. MSI status or mutations with known impact on drug sensitivity (Fig. S1) were incorporated as interaction terms in two parallel runs. Model performance was evaluated based on Pearson correlations between the actual and predicted DSS in a test set of samples. Drugs targeting BRAF V600E, IGF1R or EGFR/HER2 were best predicted with mutations as the modifying term, but the MSI model performed better across the full drug screen and was selected for further analyses (*p* = 4 × 10^–8^ by paired t-test; Fig. S20a-d). Performance of the MSI model varied considerably among drugs (median Pearson correlation 0.52, 10th-90th percentile range 0.20–0.72) and was weakly associated with the number of genes selected for each drug model (median of 20 genes; Pearson correlation 0.29, *p* = 5 × 10^–5^; Fig. S20e-g and Table S6). The best-performing model was for the mitotic inhibitor indibulin (Pearson correlation 0.84), followed by several additional chemotherapies (Fig. 6a).

The transcriptomic models successfully predicted most exceptional drug sensitivities (77.7%, *n* = 21 of 27 modeled cell line-drug pairs with unique mechanisms of action; Fig. 6b and Fig. S21a). This included cell line-drug pairs with no other proposed molecular response mechanisms, such as the chemotherapy 8-amino-adenosine in CL-34 and the proteasome inhibitor oprozomib in Co-115. Exceptional sensitivities that were not well predicted included EGFR/HER2 inhibitors in HROC309, the NAMPT inhibitor daporinad in DLD-1, the AKT inhibitor triciribine in HCA7 and HROC18, as well as two models that predicted only one of two cell lines each (encorafenib and NMS-873). These drugs were generally better modeled with mutations as the modifying term (*p* = 0.09 by paired t-test; Fig. S21b).

## Discussion

The Exceptional Responders Initiative provided important insights into the mechanisms of exceptional treatment response in cancer but included few patients with CRC [8]. This study provides in vitro support that exceptional response can be obtained in a substantial proportion of CRCs when considering a broad range of drugs and therapeutic targets. Beyond standard-of-care agents such as irinotecan and inhibitors of EGFR, HER2 and BRAF, exceptional response was obtained with additional kinase inhibitors and targeted agents, as well as chemotherapies and non-oncology drugs. Some of these drugs are under clinical development. For example, floxuridine is an analogue of the standard chemotherapy 5-fluorouracil, and patients treated by hepatic resection for metastatic CRC might have a survival benefit from floxuridine administered by hepatic arterial infusion in addition to standard systemic treatment [40]. Furthermore, repurposing of non-oncology drugs for anticancer use is an appealing strategy [41], and an epidemiological study suggested that continued use of the alcohol-aversion drug disulfiram (trade name Antabuse) after a cancer diagnosis may reduce the risk of death [42]. Addition of disulfiram to chemotherapy can also prolong the survival of patients with metastatic lung cancer but showed limited efficacy when evaluated in treatment refractory metastatic CRCs [43, 44]. Limited clinical efficacy is consistent with the common challenge that exceptional responses typically occur in the background of a low overall response rate [1]. Most drugs identified in this study had exceptional activity in only one or two cell lines each, supporting the need for predictive biomarkers and careful patient selection. However, a main limitation of the study is the preclinical design, and it is not clear whether these data can be translated into clinical responses. Furthermore, the criteria for detection of exceptional responses in patients are challenging to translate into in vitro studies. The Exceptional Responders Initiative identified responses to treatments with a low overall efficacy (population response rate less than 10%), or with an unusually long duration of response (at least 3 times the published median) [7]. These criteria identified “outlier patients” and support the rationale for outlier detection also in our in vitro study. Furthermore, an outlier approach is consistent with the realization that most new treatment subgroups in the era of precision oncology are small [5]. However, the criteria used in our study were quite stringent, to accommodate the large number and diversity of drugs investigated, and it is likely that additional exceptional responses remain to be discovered in the dataset. Consequently, the in vitro outlier approach cannot project the proportion of CRCs that are vulnerable to exceptional treatment response but provides an appropriate basis for detection of the underlying molecular mechanisms.

Molecular underpinnings were proposed for most exceptional in vitro responses, supporting the validity of the pharmacological data and approach. Molecular correlates were investigated primarily on the gene and protein expression levels, based on the strong correspondence of transcriptomic and pharmacological profiles in this study, and on the superior predictive power relative to other molecular data levels in previous studies [41, 45]. The frequency of proposed marker-drug interactions was higher than in the Exceptional Responders Initiative (approximately one-fourth of cancers) [8], which might be rationalized both by the advantage that pure cancer cell cultures can reveal clearer pharmacogenomic associations, and by the challenge that cell lines cannot recapitulate the full complexity and heterogeneity of in vivo tumor responses. Functional validation experiments confirmed target engagement of selected kinase inhibitors and supported post-treatment suppression also of markers of extended molecular mechanisms. The latter suggested that the baseline (pre-treatment) markers were also engaged in the treatment response, which is not necessarily the case for markers beyond direct therapeutic targets. This can be rationalized by the strong outlier expression of at least some of the markers, illustrating the value of using exceptional responders as treatment models and basis for biomarker discovery. A limitation of these functional investigations was that the mechanistic links between the markers and drug treatments were not investigated in detail. Instead, we prioritized validation experiments to support biomarker engagement for several of the exceptional responses.

Molecular markers of exceptional treatment response may help to identify additional patients who will benefit from the same treatment. The most prominent example of this approach is *EGFR* mutations as a basis to preselect patients with lung cancer for EGFR inhibition [46]. A challenge with some of the proposed markers in this study in this respect is that the expression profile was not sufficiently distinct in the exceptional responder cell line to justify a strong predictive value. Furthermore, the specific markers of recurrent exceptional sensitivity to the same or similar drug varied across cell lines, although they typically involved the same molecular pathway and converged on the same function. These challenges support the use of multi-marker panels, and we developed multivariable transcriptomic models that could predict most of the exceptional drug sensitivities. These prediction models also strengthen the notion that exceptional responses have a strong molecular basis, but additional validation studies are needed to confirm the predictive value in independent data sets. Notably, apoptotic regulators seemed to be recurrently involved in exceptional sensitivity to several drugs and in several cell lines. This was not confounded by the endpoint of the drug perturbation assays, which was based on cell viability rather than cell death, and experimental validation confirmed downregulation of one of the markers in response to PAK4 inhibition. Evasion of apoptosis has been termed a hallmark of treatment resistance in cancer [47], and this study suggests that apoptotic markers can pinpoint a subgroup of CRCs with particularly strong therapeutic vulnerabilities. This is in line with a previous study showing potential to predict response to chemotherapy based on early drug-induced death signaling [48].

Some of the drugs proposed in this study have been discontinued from further clinical development due to off-target effects and toxicities or limited efficacy as single agents. An illustrative example is the aurora kinase inhibitor [49]. However, aurora kinases are still attractive drug targets and ongoing efforts focus on novel inhibitors or other targeting strategies, such as degradation by proteolysis targeting chimeras (PROTACs) [50]. Our study supports targeting of aurora kinases in highly selected CRCs, even in an MSS *RAS* mutated background. Furthermore, some of the drugs and targets are considered attractive partners for combination therapy. For example, both PAK and farnesyl transferase inhibitors have in vitro synergy with KRAS G12C inhibitors [51, 52], which is of particular interest considering that KRAS G12C is an emerging treatment target in metastatic CRCs [53]. This study identified exceptional responses to PAK and farnesyl transferase inhibitors in cell lines with the *BRAF* p.V600E mutation, suggesting combination testing also with this drug target. Altogether, these data provide new hypotheses for targeting the hard-to-treat CRCs driven by aberrant MAPK pathway activity.

The main limitation of the study was the lack of clinical translation, and we consider the study a foundation for new hypotheses in precision medicine of CRC. Cell lines were used as the model system due to compatibility with the high-throughput experimental design, but validation experiments in more physiologically representative ex vivo or in vivo models will be needed to support the potential for exceptional treatment responses in tumors. Some drugs used in the standard of care for CRC were not evaluated due to their dependence on an active tumor microenvironment, such as immunotherapies and antiangiogenic agents, and the drug libraries did not include novel emerging drugs such as the mutation-specific KRAS or pan-RAS inhibitors. Furthermore, the pharmacological analyses were performed as single-agent screens and drug synergies were not evaluated. Most systemic treatments for CRC are provided as drug combinations, and these might have superior activity over single agents due to additive or synergistic effects and tumor heterogeneity. It remains unknown whether drug combinations are needed for successful clinical translation of new exceptional treatment responses in CRC.

In conclusion, this study identifies profound cancer cell vulnerabilities and shows in vitro potential to obtain exceptional treatment response to diverse drug classes and drug targets in CRC. Most exceptional responses have clear molecular mechanisms, and the study highlights the value of exceptional responders as models to discover predictive biomarkers that can potentially guide molecular prescreening and patient selection. A strength of the study was the size of the drug- and cell line panels, all representing a single cancer type. We provide a compendium of exceptional drug responses and their molecular markers in conventional CRC cell lines, and the data are made openly available as a preclinical pharmaco-transcriptomics resource.

## Materials and methods

### Cell lines

A collection of 103 CRC cell lines purchased from cell line repositories or kindly provided by collaborators were analyzed (Table S1). This included 101 unique cell lines derived from 99 patients, as well as 2 cell line derivatives (DLD-1 derived from HCT 15; WiDr derived from HT-29) and 2 cell line pairs derived from patient-matched primary and metastatic tumors (SW480 and SW620; Isreco-1 and Isreco-3). All cell lines were derived from adenocarcinomas, except three with a neuroendocrine origin (COLO 320, HROC57 and NCI-H716). Most cell lines were from primary tumors (80.0%), and the remaining from diverse metastatic sites. The cell lines were cultured in humidified incubators at 37 °C and 5% CO_2_ using medium with added fetal bovine serum, glutamine and antibiotics (Table S1). Mycoplasma testing was performed prior to experiments using the MycoAlert Mycoplasma Detection Kit (Lonza, Walkersville, MD, USA). All experiments were performed on the same passage of each cell line (median of 5 passages after procurement, range 3–9).

DNA and RNA were extracted using the AllPrep DNA/RNA/miRNA Universal Kit (Qiagen GmBH, Hilden, Germany). Authenticity and/or unique origin of all cell lines (the same passage used for experiments) has previously been confirmed by short tandem repeat profiling [21]. All cell lines have been analyzed for MSI status and sequenced for a custom panel of 20 CRC-relevant genes (Table S1). A subset of the cell lines has previously been analyzed by transcriptomic profiling (*n* = 34, 33.0%) and expression of 297 proteins and phospho-proteins on reverse-phase protein arrays (*n* = 33, 32.0%) [18].

### Gene expression analyses

Transcriptomic profiling was performed with Affymetrix Human Transcriptome 2.0 arrays (Thermo Fisher Scientific, Waltham, MA, USA). Details of data processing are described in the Supplementary Material. Transcriptomic similarity to CRC tissue was evaluated using the R package CancerCellNet (v. 0.2.0) [17]. Classification according to CMS and iCMS was performed using the R package CMScaller (v. 2.0.1) with default settings [54] and original gene template for iCMS [55]. Additional details are described in the Supplementary Material. PCA was performed using the R package FactoMineR (v. 2.11) on genes (*n* = 3,000) with the highest cross-sample 10-90th percentile range. Single-sample enrichment scores of gene sets in the KEGG Medicus collection [56] (*n* = 658, accessed from the Molecular Signatures Database) and a custom collection (*n* = 43; Table S3) were estimated using the gsva function in the R package GSVA (v. 2.0.7) [57]. A proliferative index was estimated with the R package ProliferativeIndex (v. 1.0.1) [58].

### Drug sensitivity testing

Cell lines were analyzed for sensitivity to two drug libraries using the high-throughput drug sensitivity and resistance testing platform at the Finnish Institute of Molecular Medicine (FIMM, Helsinki, Finland) [59]. The FIMM oncology collection version 4 A (FO4A, *n* = 461 drugs) was screened in 38 cell lines and version 5 A in 65 cell lines (FO5A, *n* = 528 drugs; Table S1). Most drugs (*n* = 369, 59.5%) overlapped between the two libraries, and the total number of screened drugs was 620 (Table S2). Each drug was dispensed in 384-well plates at five different concentrations over a 10,000-fold concentration range, and cells were seeded onto the preprinted plates. Cell viability was measured with the CellTiter-Glo assay after 72 h incubation (Promega, Madison, WI, USA) and rescaled to relative viability based on the median of positive (100 μmol/L benzethonium chloride) and negative control wells (0.1% DMSO). Dose response curves were fitted to the relative viability data with logistic regression using the logLogisticRegression function in the R package PharmacoGx (v. 3.0.2) [60]. This returned curve parameter estimates such as the Hill coefficient (slope), the asymptote as the drug concentration tends to infinity (E_inf), and the drug concentration at the half maximum effect (EC_50). These parameters were used as input to estimate DSS values based on the area under the dose response curves, using the R package DSS [61]. Plates with a strictly standardized mean difference of DSS below 6 were excluded from analyses (*n* = 33 of 926 plates, 3.6%). Drugs with low activity in all cell lines (maximum DSS < 7) and low variation of activity (cross-sample 10-90th percentile range < 5) were filtered from selected analyses (*n* = 294, 47.4%; Table S2).

Hierarchical clustering of DSS values based on Euclidean distances and with complete linkage was performed for drugs included in both libraries and retained after filtering (*n* = 203). Heatmap and dendrogram were generated using the heatmap.2 function with default settings in the R package gplots (v. 3.2.0). PCA of DSS values was performed for all drugs retained after filtering (*n* = 326), using missing data imputation with the imputePCA function in the R package missMDA (v.1.19).

No batch correction was performed between the two drug libraries due to overrepresentation of MSI cell lines in the FO4A screen (*p* = 0.001 by Fisher’s exact test). However, replicate drug screens (*n* = 12) of 4 cell lines were performed for quality control and showed good correlation of samples screened with both libraries and with the same library over time (Fig. S22). Hierarchical clustering showed similar activity across cell lines for drugs with the same molecular target and/or mechanism of action (Fig. S1c).

### Exceptional drug sensitivities

Exceptional drug sensitivities were identified with Tukey’s outlier statistics in the DSS matrix of 620 drugs and 103 cell lines. This method scored DSS values higher than 1.5 × the interquartile range above the 3rd quartile as outliers (Q3 + 1.5 × [Q3-Q1]). Outlier detection was performed for each row (drug) and column (cell line) of the matrix, and exceptional drug sensitivities were required to be scored as outliers in both dimensions. This requirement was implemented to account for variation in the distribution of DSS values of individual drugs and cell lines, and to avoid false scoring of drugs with high activity in many samples and of samples with high sensitivity to many drugs, as illustrated in Fig. S6. The DSS matrix had near complete measurements for drugs overlapping between the two libraries (*n* = 369), and drugs that were unique to each library were analyzed in a subset of the cell lines (*n* = 92 drugs in 36–38 cell lines and *n* = 159 drugs in 61–65 cell lines). No filtering based on drug activity was performed prior to outlier detection. Notably, the distribution of DSS values showed a heavy right-sided tail in all cell lines and the assumption of a normal distribution among non-outliers was not met (by Shapiro–Wilk tests). Furthermore, outlier detection was performed without multiple testing correction. The risk of false positives was instead accounted for by orthogonal validation approaches, including analyses of an external dataset, detection of molecular underpinnings, and transcriptomic predictions of exceptional drug sensitivities.

### External validation dataset

The GDSC2 dataset [15] was downloaded from Cell Model Passports and compared with overlapping cell lines (*n* = 34) and drugs (*n* = 130) in the in-house dataset. Additional details are described in the Supplementary Material.

### Validation experiments with drug treatment and Western blotting

Cell lines (*n* = 7) selected based on exceptional drug sensitivities (criteria described in the Results) were treated with relevant drugs (total *n* = 7) at five different concentrations and vehicle control before Western blotting of selected antibodies as described in the Supplementary Material and Table S5.

### Transcriptomic prediction of drug sensitivity

Transcriptomic prediction models of drug sensitivity were constructed using MADMMplasso (v. 1.0.0) [39] on the common set of drugs between the two libraries after filtering based on activity (*n* = 203; Table S2). Modeling was performed twice, using either MSI status or selected gene mutations for potential interactions (labelled the “MSI model” and “mutation model”, respectively). Additional details are described in the Supplementary Material.

### Further statistical analyses

All further statistical analyses were performed with R version 4.4.2. All statistical tests were two-sided and *p*-values < 0.05 were considered significant. Box plots were drawn with the median as the center line, boxes represent the interquartile range, and whiskers represent 1.5 × the interquartile range above the 75th percentile or below the 25th percentile. Circos plots were generated with the package circlize (v. 0.4.16), alluvial plots with the package alluvial (v. 0.1–2), and correlation matrix with the package ggcorrplot (v. 0.1.4.1).

## Supplementary Information

Supplementary Material 1. Fig. S1 to S22 and Supplementary Methods

Supplementary Material 2. Tables S1 to S7

## Data Availability

The transcriptomics dataset supporting the conclusions of this article were deposited into the Gene Expression Omnibus database under accession number GSE309172 and are available at the following URL: https://www.ncbi.nlm.nih.gov/geo/query/acc.cgi?acc=GSE309172. The drug sensitivity dataset (DSS values) is available within the article and its Supplementary Information files (Table S7). Cell line authentication data (short tandem repeat profiles) and mutation data have previously been published and are available in Supplementary Tables S2 and S9 of the corresponding article at the following URL: https://static-content.springer.com/esm/art%3A10.1038%2Fs41389-026-00599-0/MediaObjects/41389_2026_599_MOESM2_ESM.xlsx [21]. All data processing and analyses were performed with published software packages or computer code and have been described and cited in the Methods and/or Results sections of this article. No custom code was developed in the study.
