# Supplementary material for: Exceptional treatment responses and molecular markers among 103 colorectal cancer cell lines
Source: Mol Biomed. 2026 Aug 25;7:150. doi: 10.1186/s43556-026-00551-z (PMC13507031; doi:10.1186/s43556-026-00551-z)
Supplement: Supplementary file 1 [file 43556_2026_551_MOESM1_ESM.pdf]

Supplementary Material 1 for:

Exceptional treatment responses and molecular markers among 103 colorectal cancer cell lines

Anita Sveen[1,2], Jakob M. Stenersen[1,2], Theophilus Quachie Asenso[3], Solveig K. Klokkerud[1], Christian Kranjec[1], Ina A. Eilertsen[1], Bjarne Johannessen[1], Edward Leithe[1], Kushtrim Kryeziu[1], Manuela Zucknick[3,4], Ragnhild A. Lothe[1,2]

[1]Department of Molecular Oncology, Institute for Cancer Research, Oslo University Hospital, Oslo, Norway; [2]Institute of Clinical Medicine, Faculty of Medicine, University of Oslo, Oslo, Norway; [3]Oslo Centre for Biostatistics and Epidemiology, Department of Biostatistics, University of Oslo, Oslo, Norway; [4]Oslo Centre for Biostatistics and Epidemiology, Oslo University Hospital, Oslo, Norway

Corresponding author: Anita Sveen, Department of Molecular Oncology, Institute for Cancer Research, Oslo University Hospital, P.O. Box 4953 Nydalen, NO-0424 Oslo, Norway. E-mail address: anita.sveen@medisin.uio.no. ORCID: 0000-0001-8219-6251.

Table of contents

Supplementary Figures 3

Fig. S1 Drug sensitivities according to genomic aberrations 3

Fig. S2 Exceptional drug activities with flat dose-response curves 5

Fig. S3 Drugs with exceptional activity among CRC cell lines (part 1) 6

Fig. S4 Drugs with exceptional activity among CRC cell lines (part 2) 7

Fig. S5 Validation of previously proposed outlier drug sensitivities and molecular targets 8

Fig. S6 Previously proposed strong drug activities not scored as exceptional 9

Fig. S7 CRC cell lines with exceptional drug sensitivities 10

Fig. S8 Correspondence of drug sensitivities in the in-house and GDSC2 datasets 11

Fig. S9 Molecular underpinnings of exceptional sensitivity to SN-38, the active metabolite of the chemotherapy irinotecan 12

Fig. S10 Molecular underpinnings of exceptional sensitivity to inhibitors of the MAPK pathway ... 13

Fig. S11 Molecular underpinnings of exceptional sensitivity to a BET-targeting PROTAC and SMAC mimetics 14

Fig. S12 Molecular underpinnings of exceptional sensitivity to a PAK and pan-BCL2 family inhibitor 15

Fig. S13 Molecular underpinnings of exceptional sensitivity to estrogen receptor modulators or antagonists 16

Fig. S14 Molecular underpinnings of exceptional sensitivity to floxuridine, an analog to the chemotherapy 5-fluorouracil 17

Fig. S15 Molecular underpinnings of exceptional sensitivity to EGFR/HER2 inhibitors 18

Fig. S16 Molecular underpinnings of exceptional sensitivity to a proteasome inhibitor, HIF1A inhibitor, and the chemotherapy eribulin 19
Fig. S17 Molecular underpinnings of exceptional sensitivity to a multi-kinase, NAMPT, and CHEK1 inhibitor 20
Fig. S18 Molecular underpinnings of exceptional sensitivity to a p97/VCP inhibitor and ionophore 21
Fig. S19 Micrographs of cell cultures after drug treatment in validation experiments 22
Fig. S20 Performance and feature selection for transcriptomic predictions models of drug sensitivity using MADMMplasso 23
Fig. S21 Predicted and actual DSS of drugs with exceptional activity 25
Fig. S22 Correlations of replicate drug screens 26
Supplementary Methods 27
Gene expression analyses 27
External validation dataset 27
Validation experiments with drug treatment and Western blotting 27
Transcriptomic prediction of drug sensitivity 28

Supplementary Figures

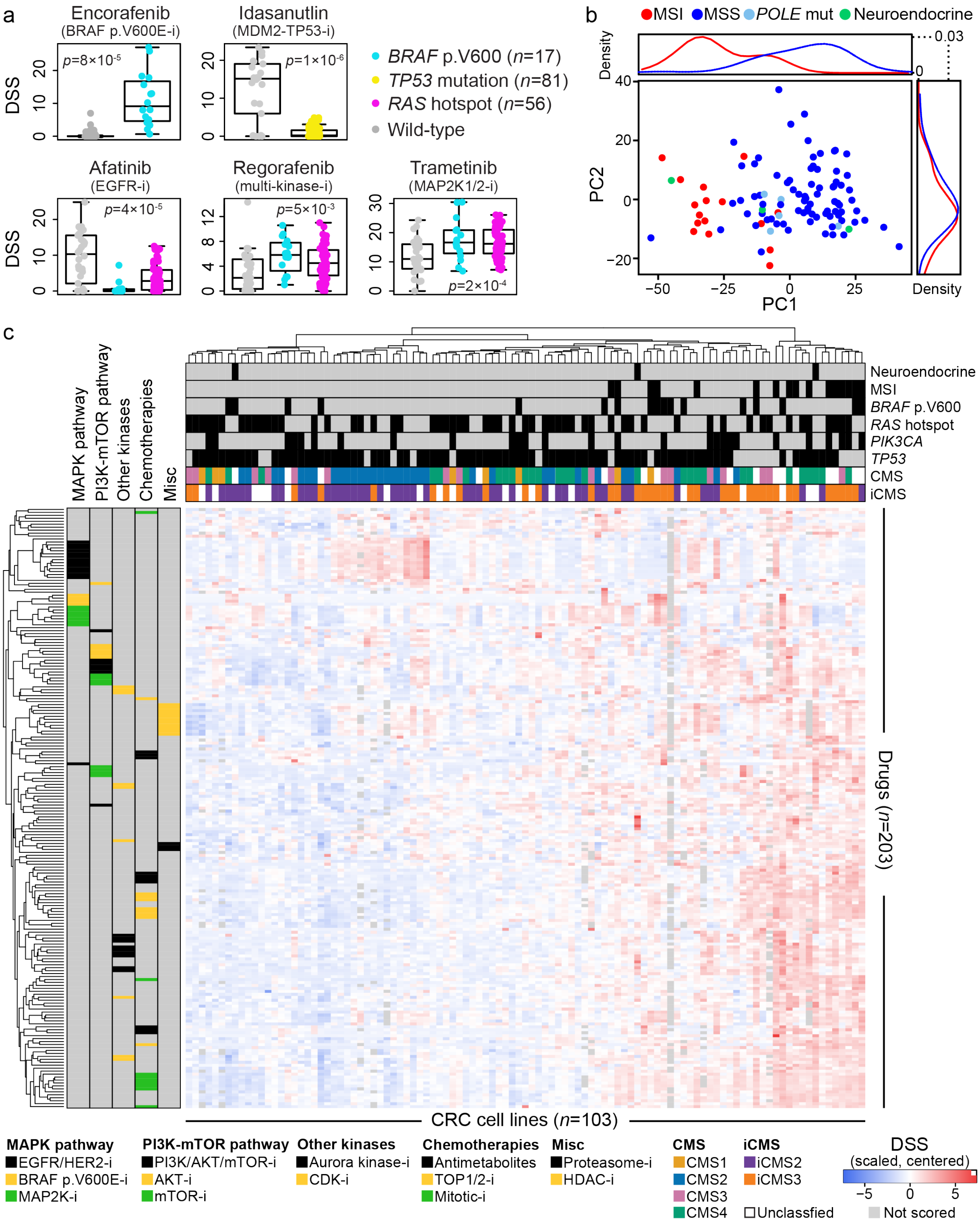

Fig. S1 Drug sensitivities according to genomic aberrations

(**a**) Boxplots of DSS values of five selected drugs according to the mutation status of cell lines (total *n* = 103) as specified. *RAS* hotspot mutations include *KRAS*/*NRAS* p.G12, p.G13, p.Q61, p.K117, and p.A146. *p*-values are from Welch's t-test of mutated versus wild-type cell lines. (**b**) PCA plot of cell lines based on DSS values (*n* = 326 drugs retained after filtering). Cell lines are colored as indicated.

The density plots on the top and right show the distribution of PC1 and PC2 values, respectively, according to MSI status of the cell lines. (**c**) Hierarchical clustering of cell lines and drugs (*n* = 203; included in both libraries and retained after filtering) based on DSS values centered to a mean of 0 and scaled to a standard deviation of 1 across samples. Dendrograms are based on Euclidean distance and complete linkage analysis. Annotation bars on the left indicate drugs (inhibitors, i) grouped according to molecular target and/or mechanism of action. Annotation bars on the top indicate selected molecular features of each cell line, with black boxes denoting MSI and mutations of the respective genes. Other color codes are specified below the heatmap.

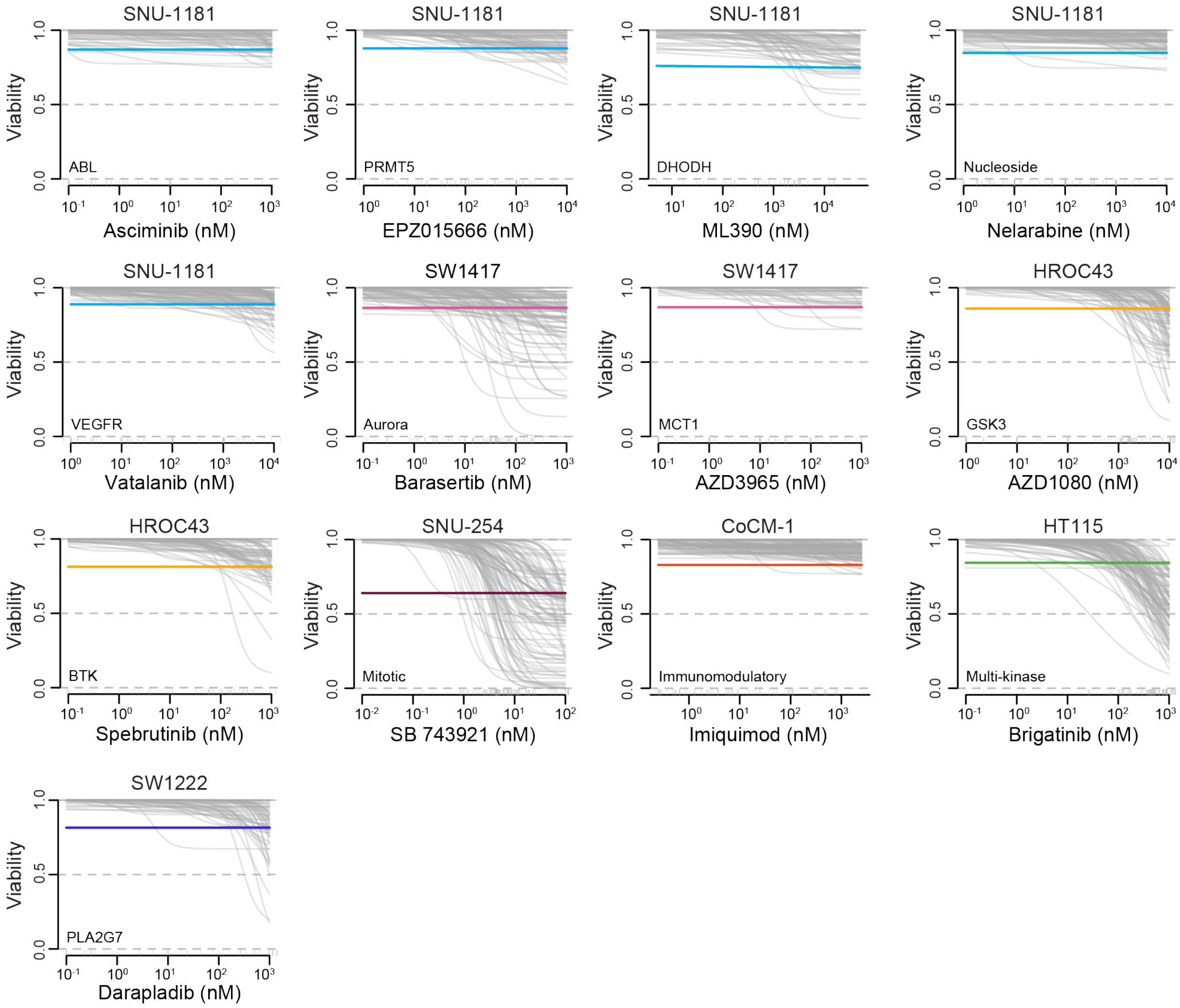

Fig. S2 Exceptional drug activities with flat dose-response curves

Dose-response plots of 13 drugs with exceptional activity in the highlighted cell lines based on outlier DSS values, but with flat dose-response curves indicating low-confidence data. Each curve represents a cell line. Drug concentrations (horizontal axes) are plotted in nanomolar (nM) and on log-10 scale. The highlighted cell lines are specified above each plot. The mechanism of action of each drug is indicated.

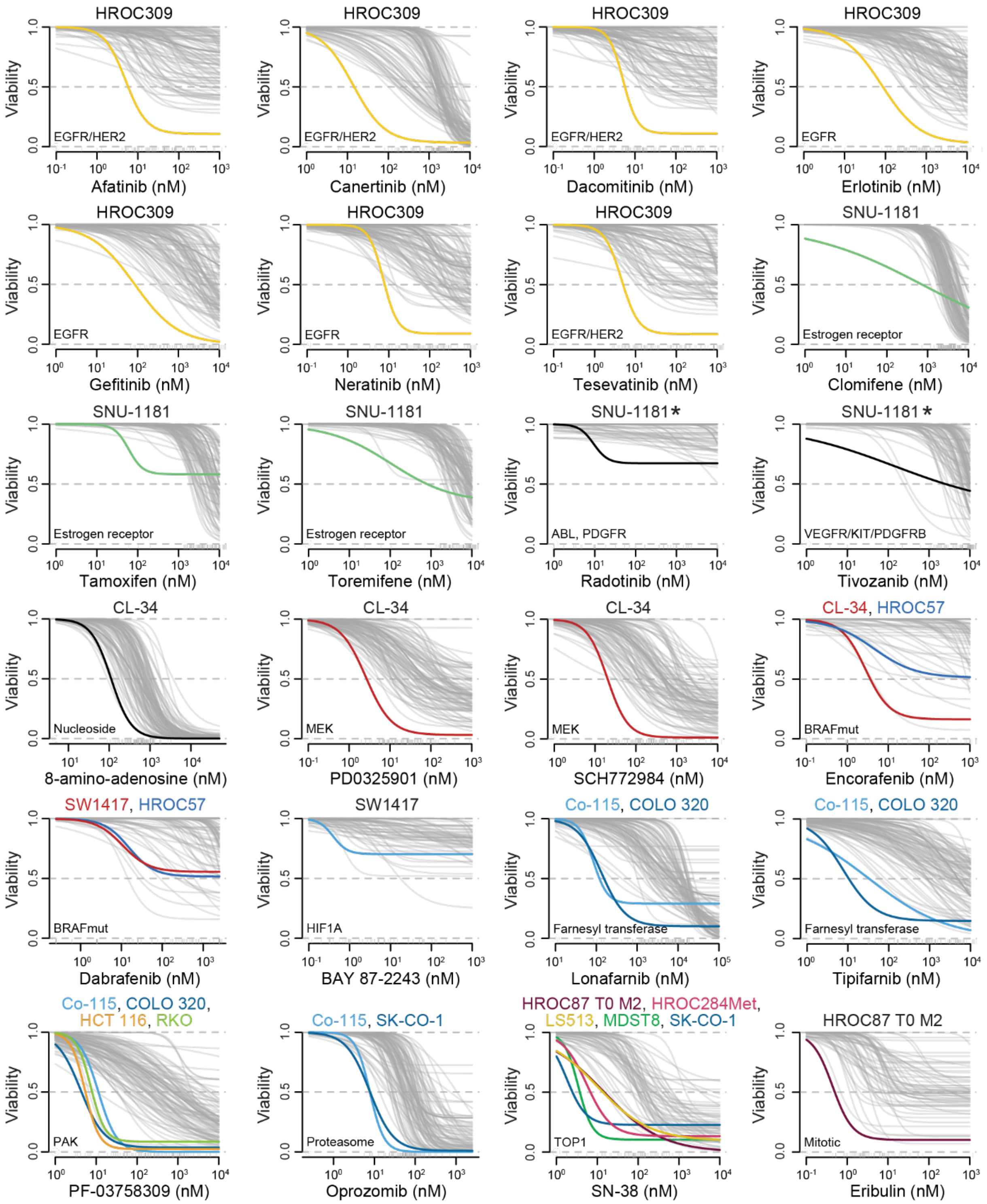

Fig. S3 Drugs with exceptional activity among CRC cell lines (part 1)

Dose-response plots of drugs (*n* = 24 of totally 49; the rest are shown in Fig. S4) with exceptional activity in at least one cell line. Each curve represents a cell line. Cell lines with exceptional sensitivity are highlighted in colors other than gray and specified above each plot. The plots are ordered according to the order of cell lines in Fig. 2b. Drug concentrations (horizontal axes) are plotted in nanomolar (nM) and on log-10 scale. The mechanism of action of each drug is indicated.

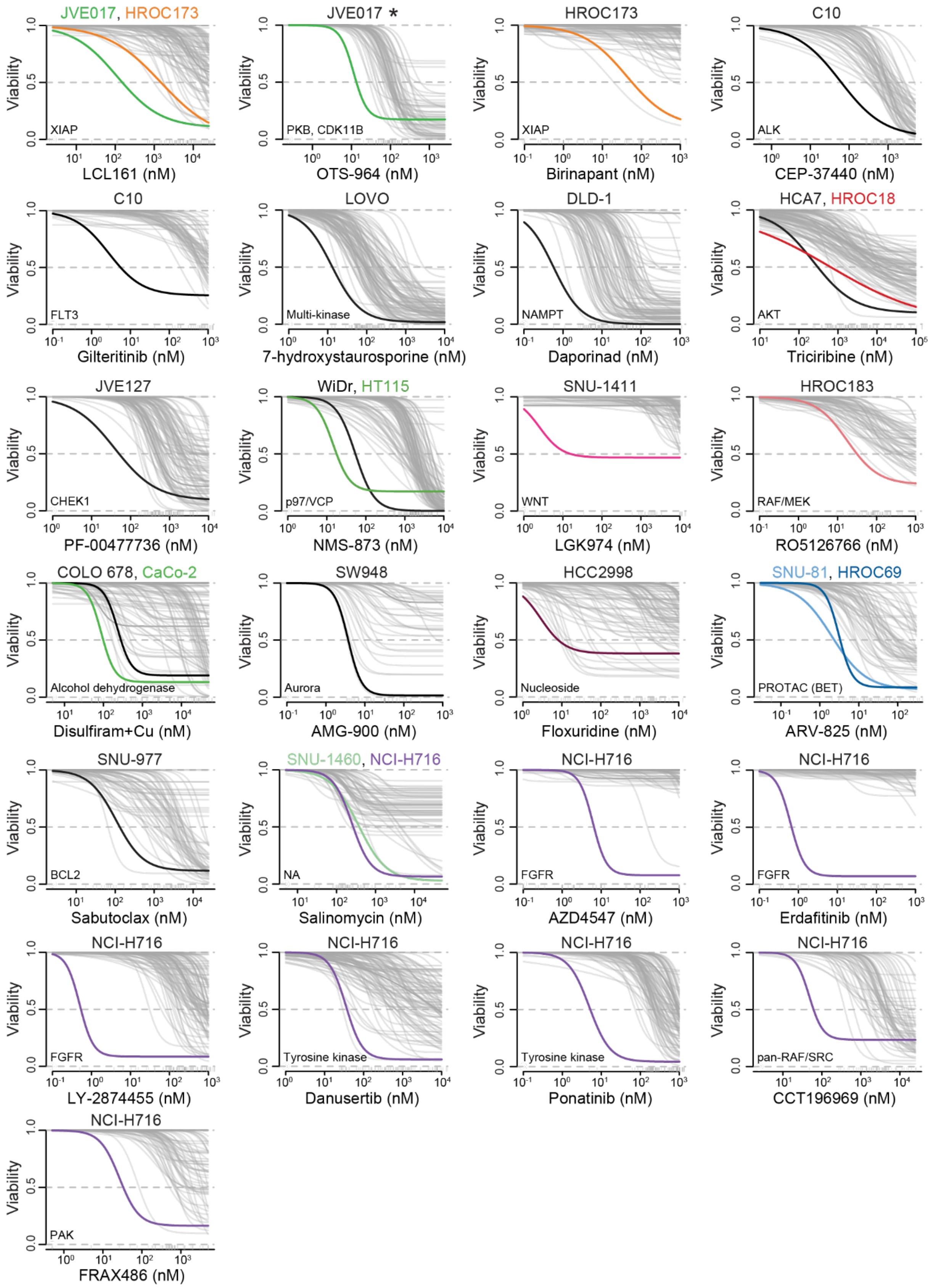

Fig. S4 Drugs with exceptional activity among CRC cell lines (part 2)

Dose-response plots of drugs (*n* = 25 of totally 49; the rest are shown in Fig. S3) with exceptional activity in at least one cell line. Plot annotations are as described in the legend of Fig. S3.

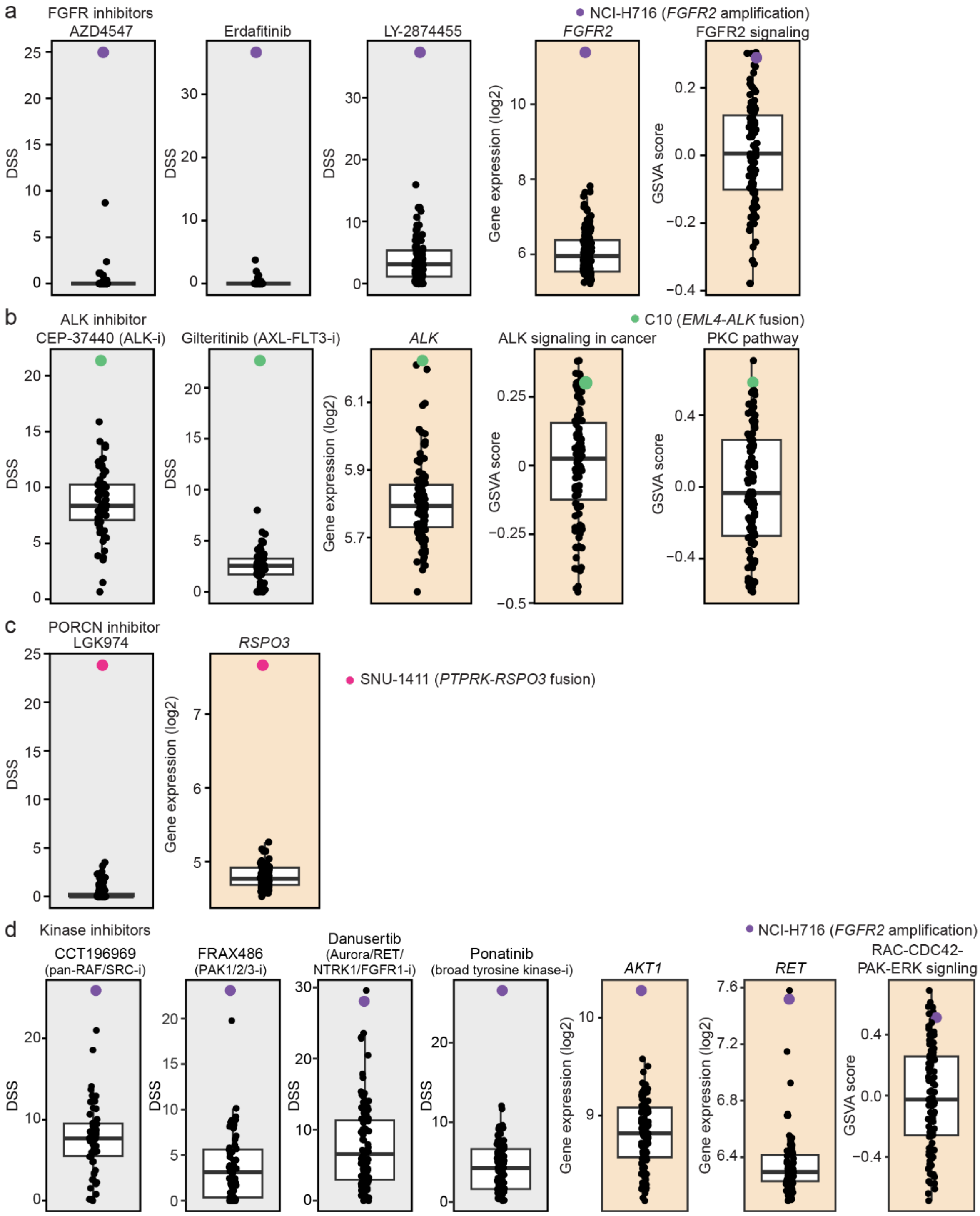

Fig. S5 Validation of previously proposed outlier drug sensitivities and molecular targets

Box plots of DSS values (gray background) of (**a**) three FGFR inhibitors, (**b**) an ALK inhibitor and AXL-FLT3 inhibitor, (**c**) a PORCN inhibitor, and (**d**) kinase inhibitors among cell lines. Cell lines with exceptional sensitivity are highlighted in purple (NCI-H716), green (C10), and pink (SNU-1411). Box plots with orange background show gene expression values of the respective drug targets or single-sample enrichment scores (GSVA scores) of relevant gene sets/pathways. High gene expression levels and enrichment scores corresponded with known mutations (amplification or fusions) in the respective drug targets and cell lines as indicated. Panels a-c show cell line-drug pairs that are described in previous publications, and panel d shows new associations for the cell line from panel a.

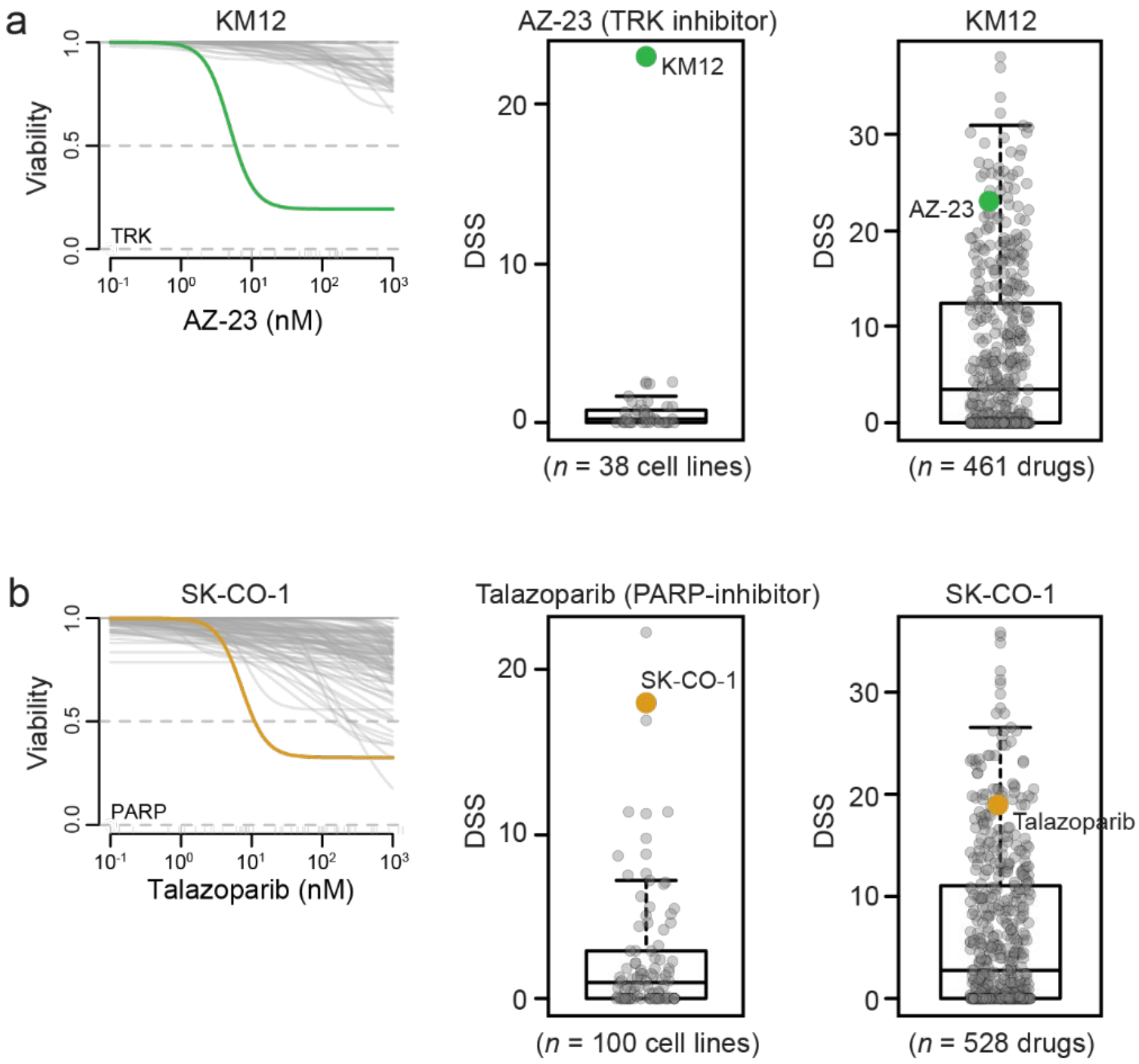

Fig. S6 Previously proposed strong drug activities not scored as exceptional

Dose-response plots (left panel) of (**a**) the TRK inhibitor AZ-23 and (**b**) the PARP inhibitor talazoparib. Each curve represents a cell line and KM12 and SK-CO-1 are marked in green and yellow in the respective plots. Drug concentrations (horizontal axes) are plotted in nanomolar (nM) and on log-10 scal1e. Box plots show DSS values of the two drugs in all cell lines screened (middle panel; drug-wise analyses) or of all screened drugs in the two cell lines (right panel; sample-wise analyses), as indicated. The two cell lines were scored as outlier samples for the respective drugs (middle panel), but the two drugs did not have outlier activity in the respective samples (right panel), and the cell line-drug pairs were therefore not scored as exceptional sensitivities.

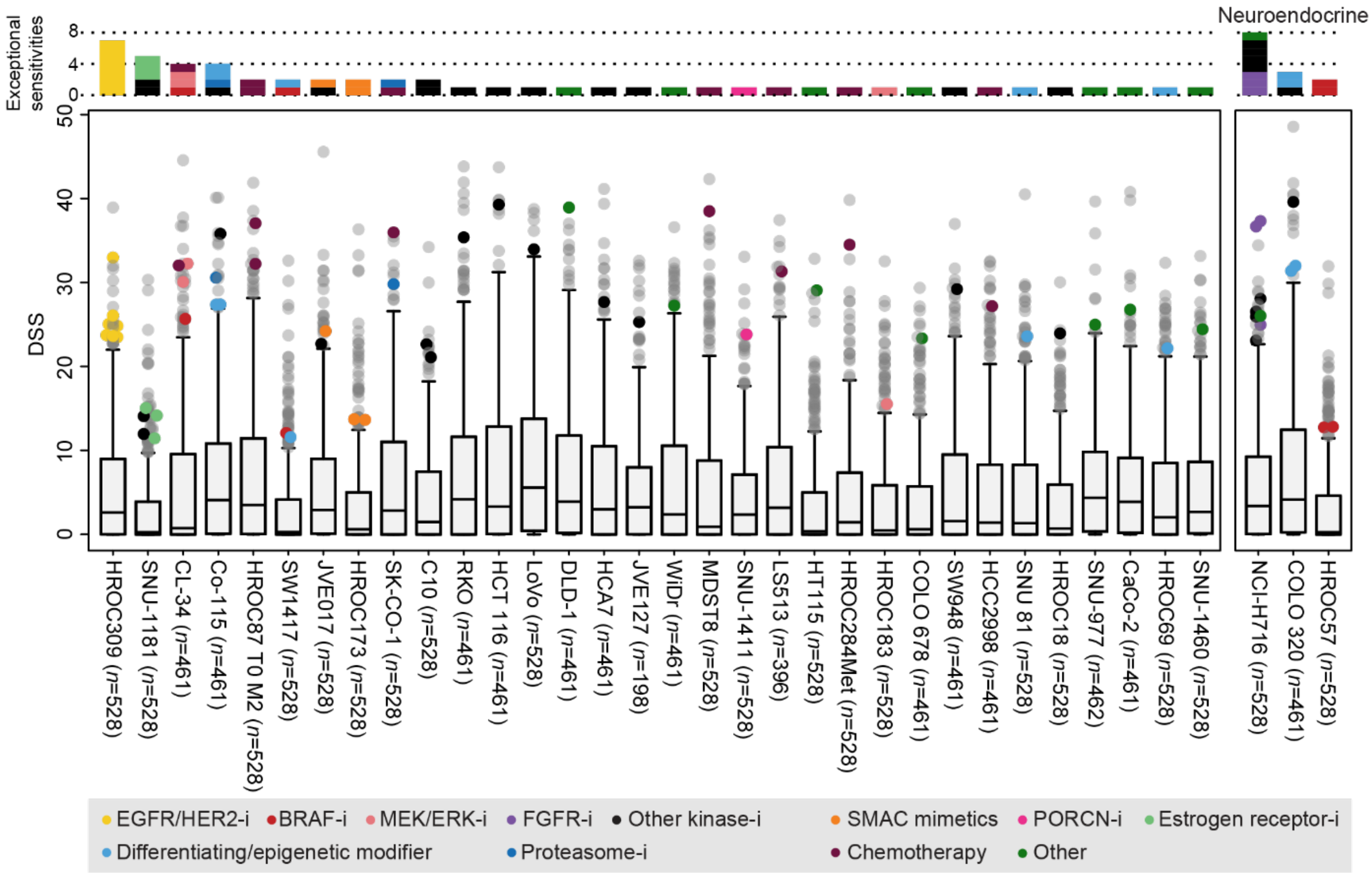

Fig. S7 CRC cell lines with exceptional drug sensitivities

Box plot of DSS values of all screened drugs in each cell line (*n* = 35) with at least one exceptional sensitivity. Cell lines are ranked in the same order as in Fig. 2b (bar plot copied above) and according to a decreasing number of exceptional drug sensitivities, with the three neuroendocrine cell lines plotted last. Drugs with exceptional activity are highlighted with colors according to drug class, as indicated in the panel below.

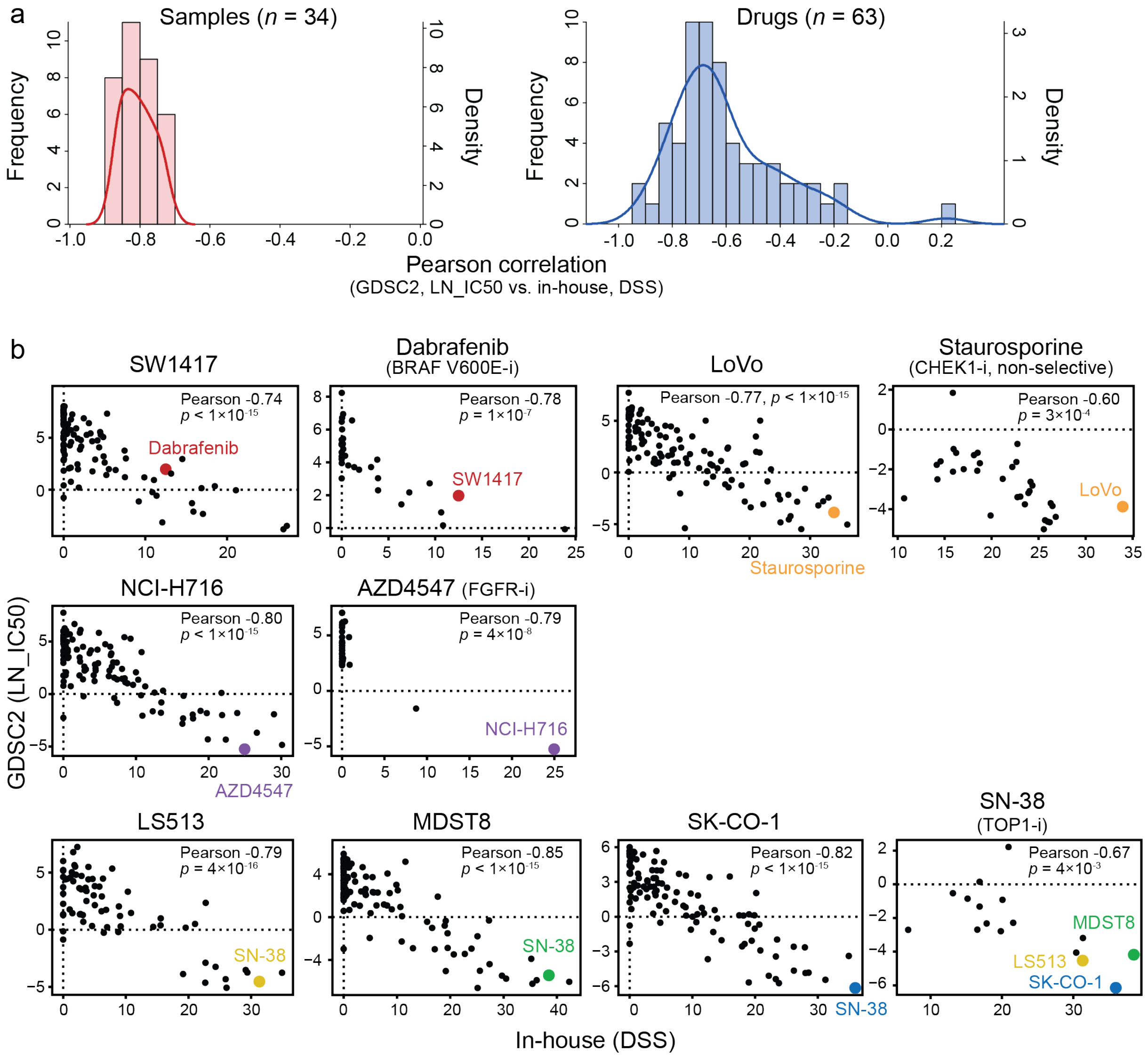

Fig. S8 Correspondence of drug sensitivities in the in-house and GDSC2 datasets

(**a**) Histograms with density curves of Pearson correlation coefficients of drug sensitivity estimates between the GDSC2 (natural logarithm of the fitted IC50 value, LN_IC50) and in-house datasets (DSS values) for overlapping samples (left) and drugs (right). The sample-wise analyses (left) included all overlapping drugs (n = 130) while the drug-wise analyses (right) were filtered to exclude drugs with frequent missing values and low variation of activity across samples. (**b**) Scatterplots of drug sensitivity estimates for the GDSC2 and in-house datasets for overlapping cell lines and drugs with exceptional sensitivities. The cell line-drug pairs with exceptional sensitivity or activity are highlighted with corresponding colors. There were 3 cell lines with exceptional sensitivity to the drug SN-38.

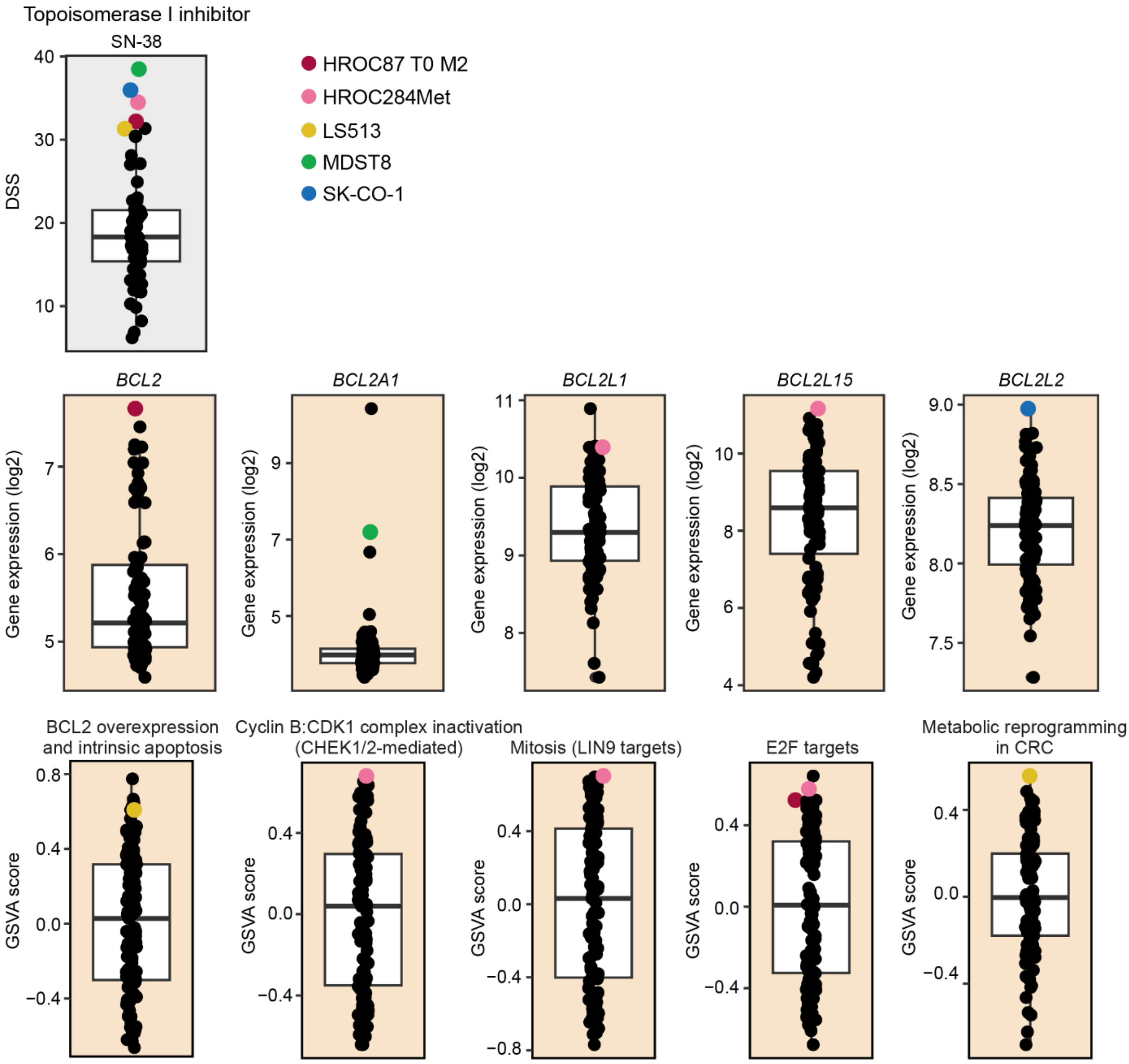

Fig. S9 Molecular underpinnings of exceptional sensitivity to SN-38, the active metabolite of the chemotherapy irinotecan

Box plot of DSS values (upper panel: gray background) of the topoisomerase I inhibitor SN-38 among cell lines. Five cell lines with exceptional sensitivity are highlighted in colors as indicated. Box plots with orange background show gene expression values of members of the BCL2 family of apoptotic regulators (middle panel) or single-sample enrichment scores (GSVA scores) of gene sets related to apoptosis or other relevant pathways (lower panel).

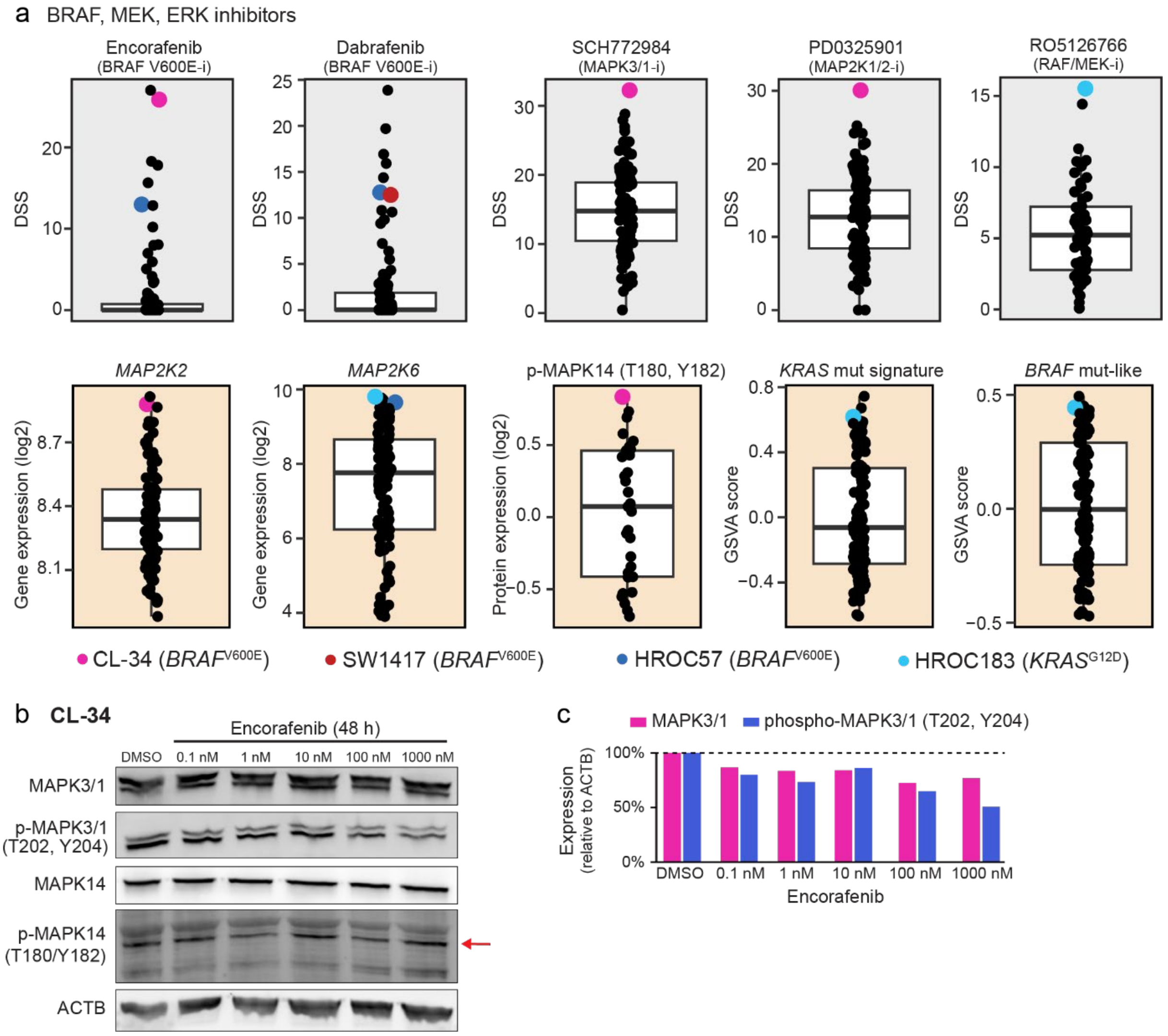

Fig. S10 Molecular underpinnings of exceptional sensitivity to inhibitors of the MAPK pathway

(**a**) Box plots of DSS values of five kinase inhibitors (i) of the MAPK pathway among cell lines (upper panel: gray background). Four cell lines with exceptional sensitivity are highlighted in colors as indicated. Box plots with orange background (lower panel) show gene or phospho-protein expression values of components of the MAPK pathway or single-sample enrichment scores (GSVA scores) of relevant gene sets/pathways. Protein expression values are median centered and phosphorylation sites are specified. (**b**) Western blots of CL-34 cells after treatment with DMSO (control) or five different concentrations of encorafenib. Samples originate from the same experiment and the gel/blot with MAPK3/1 and MAPK14 was processed in parallel to phospho-MAPK3/1 and phospho-MAPK14. ACTB was used as loading control on all blots and illustrated for the MAPK3/1 blot. Blots were run once per sample and experimental condition. The band for phospho-MAPK14 is indicated with a red arrow, and other bands on this blot are noise. (**c**) Quantification of Western blots in panel b relative to corresponding ACTB levels from each blot and centered based on expression after DMSO treatment. MAPK14 and phospho-MAPK14 were not quantified due to the noise in the blots and no apparent change in phosphorylation levels after drug treatment.

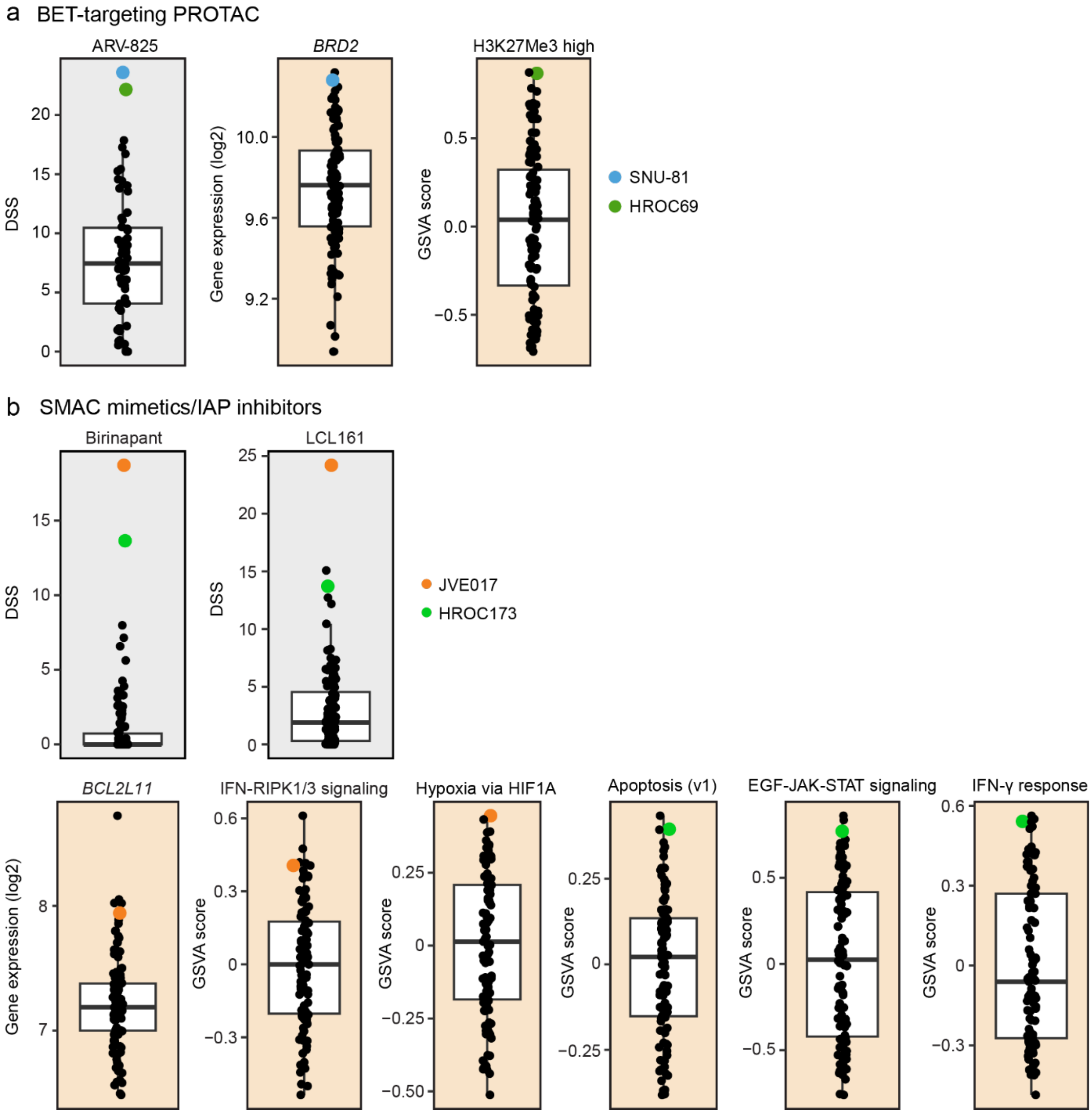

Fig. S11 Molecular underpinnings of exceptional sensitivity to a BET-targeting PROTAC and SMAC mimetics

(**a**) Box plot of DSS values (gray background) of a proteolysis-targeting chimera (PROTAC) targeting bromodomain and extra-terminal (BET) family proteins among cell lines. Two cell lines with exceptional sensitivity are highlighted in colors as indicated. Box plots with orange background show gene expression values of the target gene *BRD2*, or single-sample enrichment scores (GSVA scores) of a gene set related to histone methylation marks (H3K27Me3). (b) Box plots of DSS values of two SMAC mimetics, gene expression values of a BCL2 family member of apoptotic regulators, or GSVA scores of gene sets related to apoptosis, interferon-γ signaling or hypoxia. Two cell lines with exceptional sensitivity are highlighted in colors as indicated.

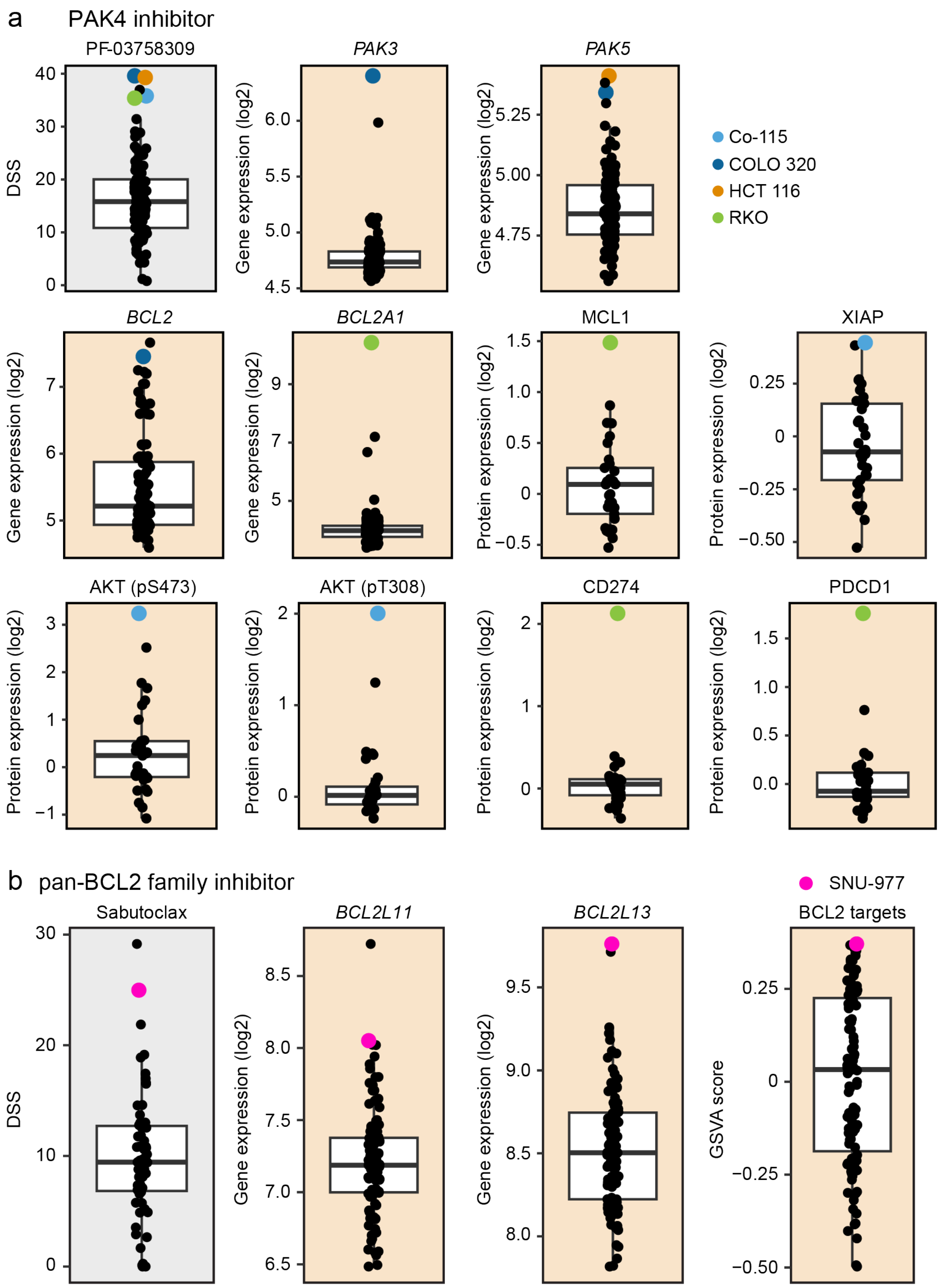

Fig. S12 Molecular underpinnings of exceptional sensitivity to a PAK and pan-BCL2 family inhibitor

(**a**) Box plot of DSS values (gray background) of a PAK inhibitor among cell lines. Four cell lines with exceptional sensitivity are highlighted in colors as indicated. Box plots with orange background show gene or (phospho-)protein expression values of drug targets or markers of extended molecular response mechanisms. Protein expression values are median centered and phosphorylation sites of AKT are specified. (**b**) Box plot of DSS values of the pan-BCL2 family inhibitor sabutoclax, gene expression of BCL2 family members, or single-sample enrichment scores (GSVA scores) of a gene set of BCL2 target activity. One cell line with exceptional sensitivity is highlighted in color as indicated.

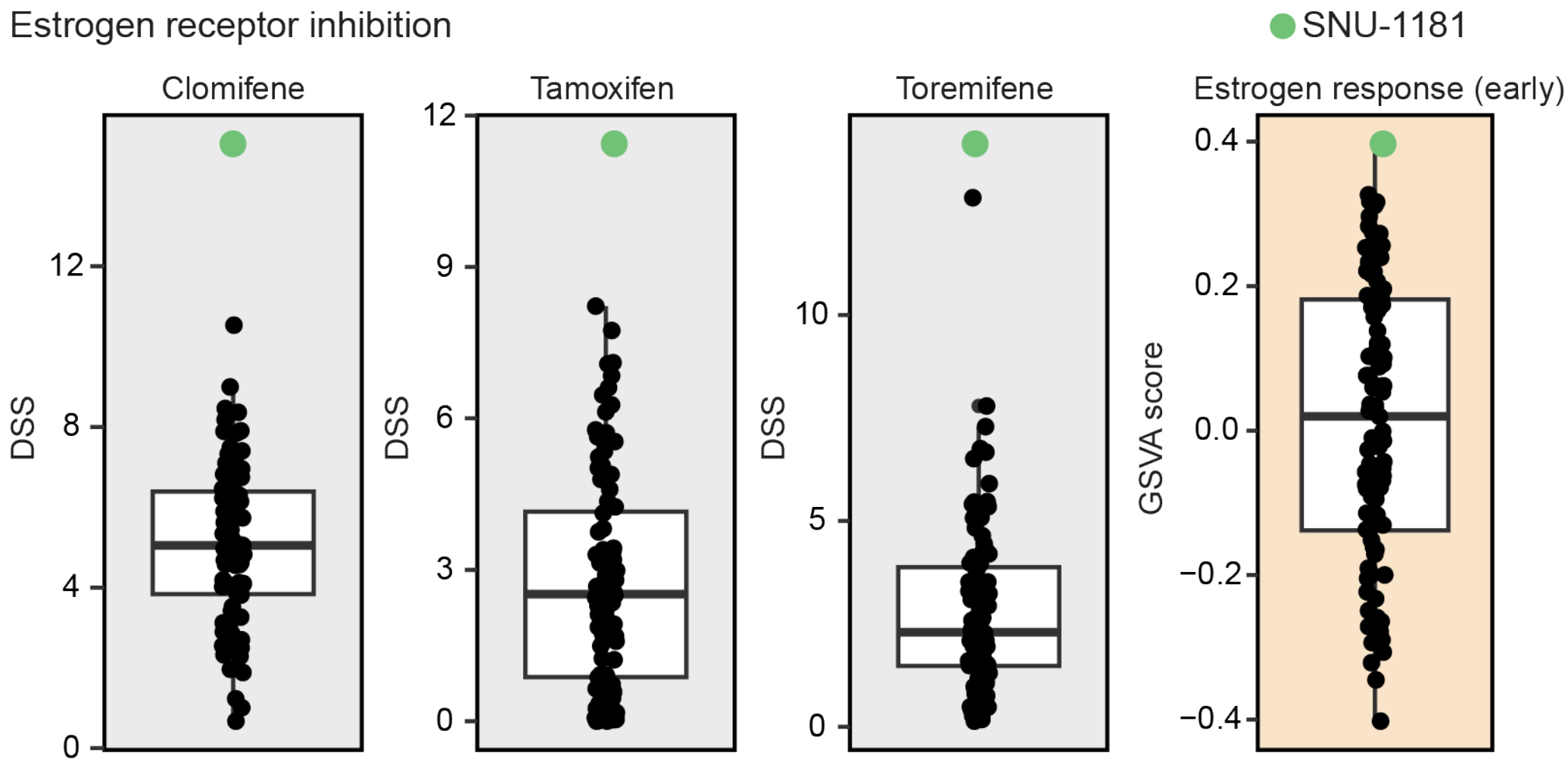

Fig. S13 Molecular underpinnings of exceptional sensitivity to estrogen receptor modulators or antagonists

Box plots of DSS values (gray background) of estrogen receptor modulators or antagonists among cell lines. One cell line with exceptional sensitivity is highlighted in color as indicated. Box plot with orange background show single-sample enrichment scores (GSVA scores) of a gene set of early estrogen response.

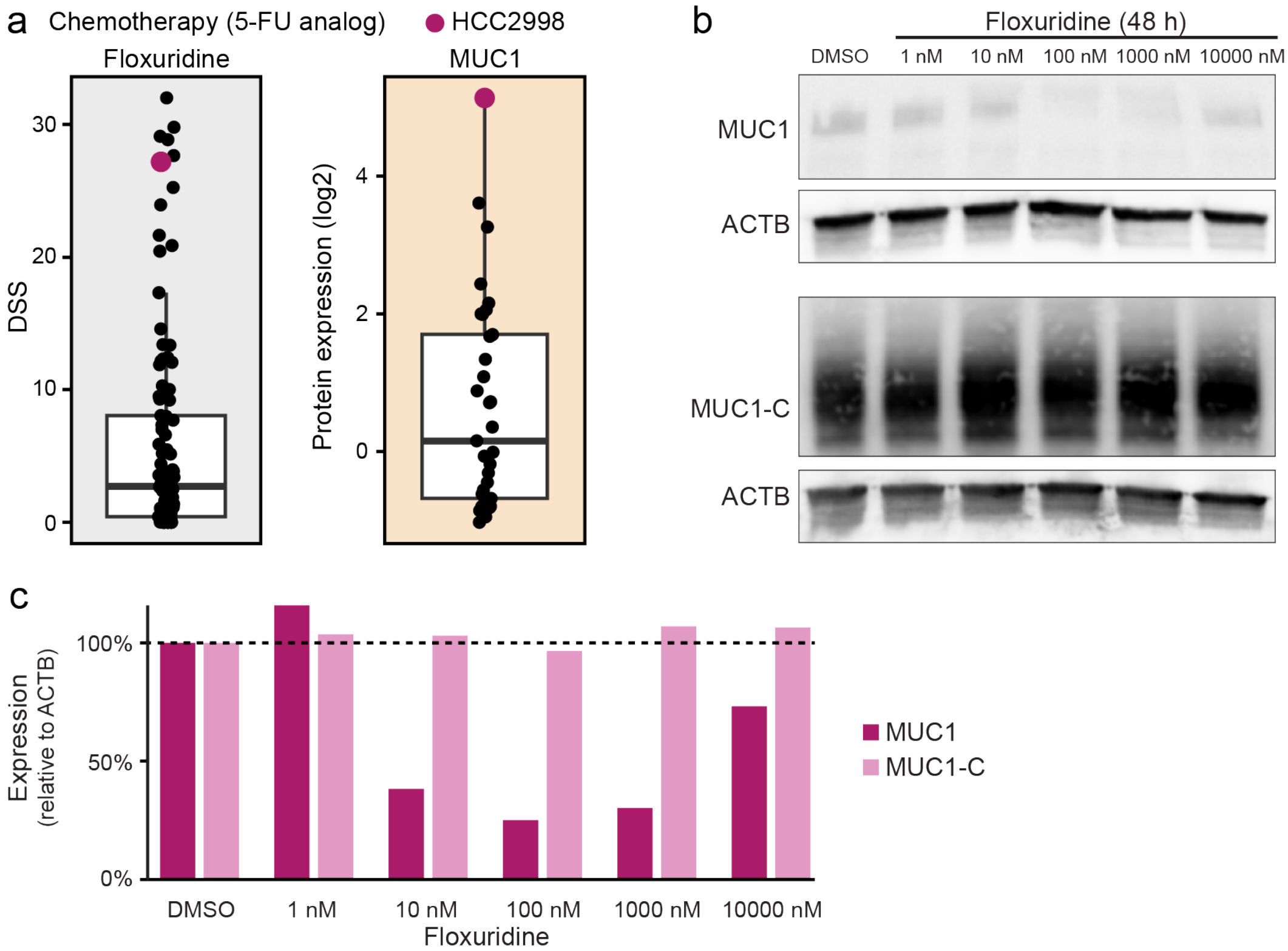

Fig. S14 Molecular underpinnings of exceptional sensitivity to floxuridine, an analog to the chemotherapy 5-fluorouracil

(**a**) Box plot of DSS values of the chemotherapy floxuridine (gray background) and protein expression values of MUC1 (orange background) among cell lines. One cell line with exceptional sensitivity is highlighted in color as indicated. (**b**) Western blots of HCC2998 cells after 48 h treatment with DMSO (control) or five different concentrations of floxuridine. Samples originate from the same experiment and the gel/blot with MUC1 was processed in parallel to MUC1-C (transmembrane C-terminal subunit). ACTB was used as loading control on both blots. Blots were run once per sample and experimental condition. (**c**) Quantification of Western blots in panel b relative to ACTB from each blot and centered based on expression after DMSO treatment.

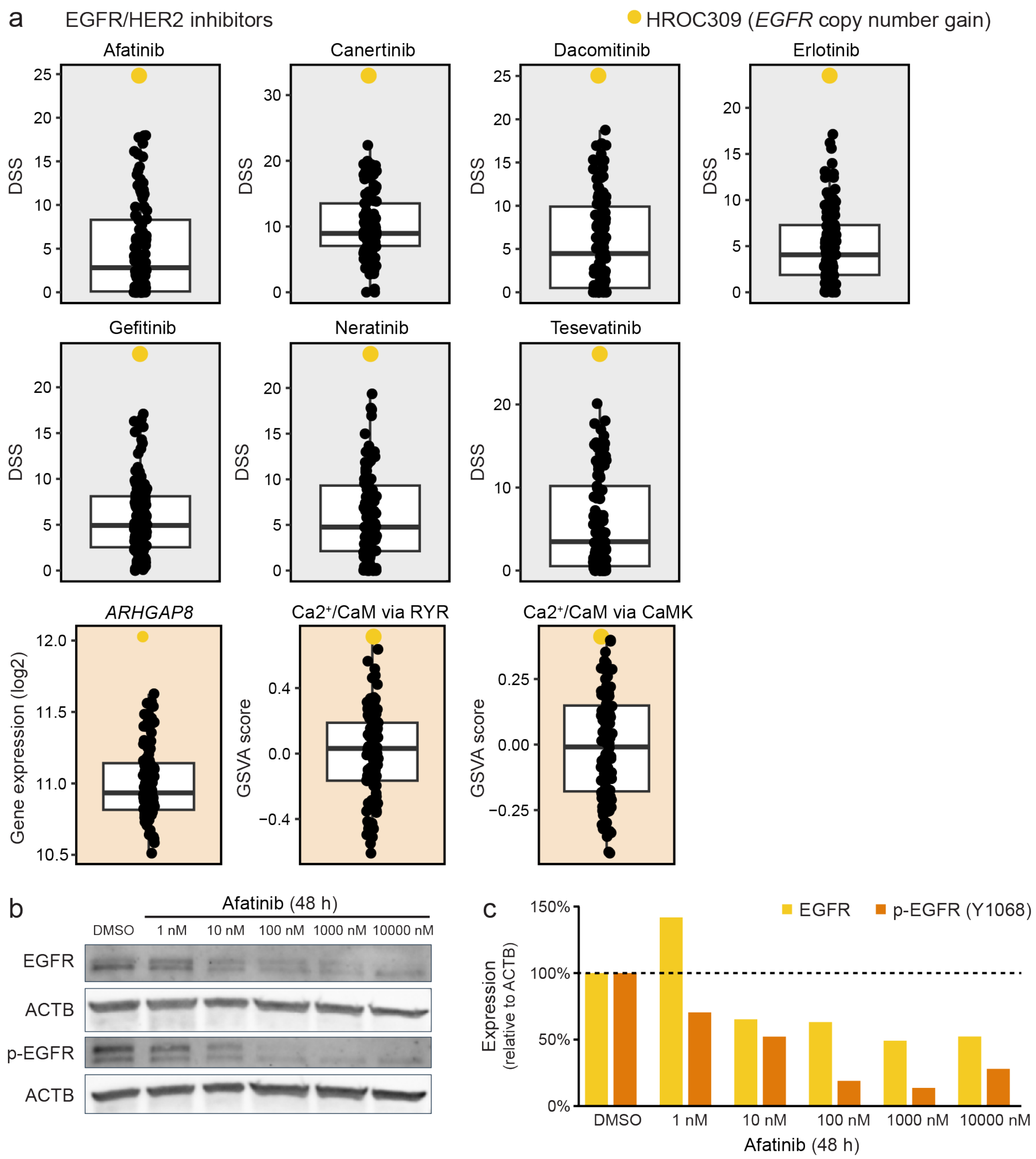

Fig. S15 Molecular underpinnings of exceptional sensitivity to EGFR/HER2 inhibitors

(**a**) Box plot of DSS values of seven EGFR/HER2 inhibitors among cell lines (gray background). One cell line with exceptional sensitivity is highlighted in color as indicated. Box plots with orange background show gene expression values of *ARHGAP8*, or single-sample enrichment scores (GSVA scores) of gene sets related to intracellular signals downstream of the Ca^{2+} sensoring protein Calmodulin. (**b**) Western blots of HROC309 cells after treatment with DMSO (control) or five different concentrations of afatinib. Samples originate from the same experiment and the gel/blot with EGFR was processed in parallel to phospho-EGFR. ACTB was used as loading control on both blots. Blots were run once per sample and experimental condition. (**c**) Quantification of Western blots in panel b relative to ACTB from each blot and centered based on expression after DMSO treatment.

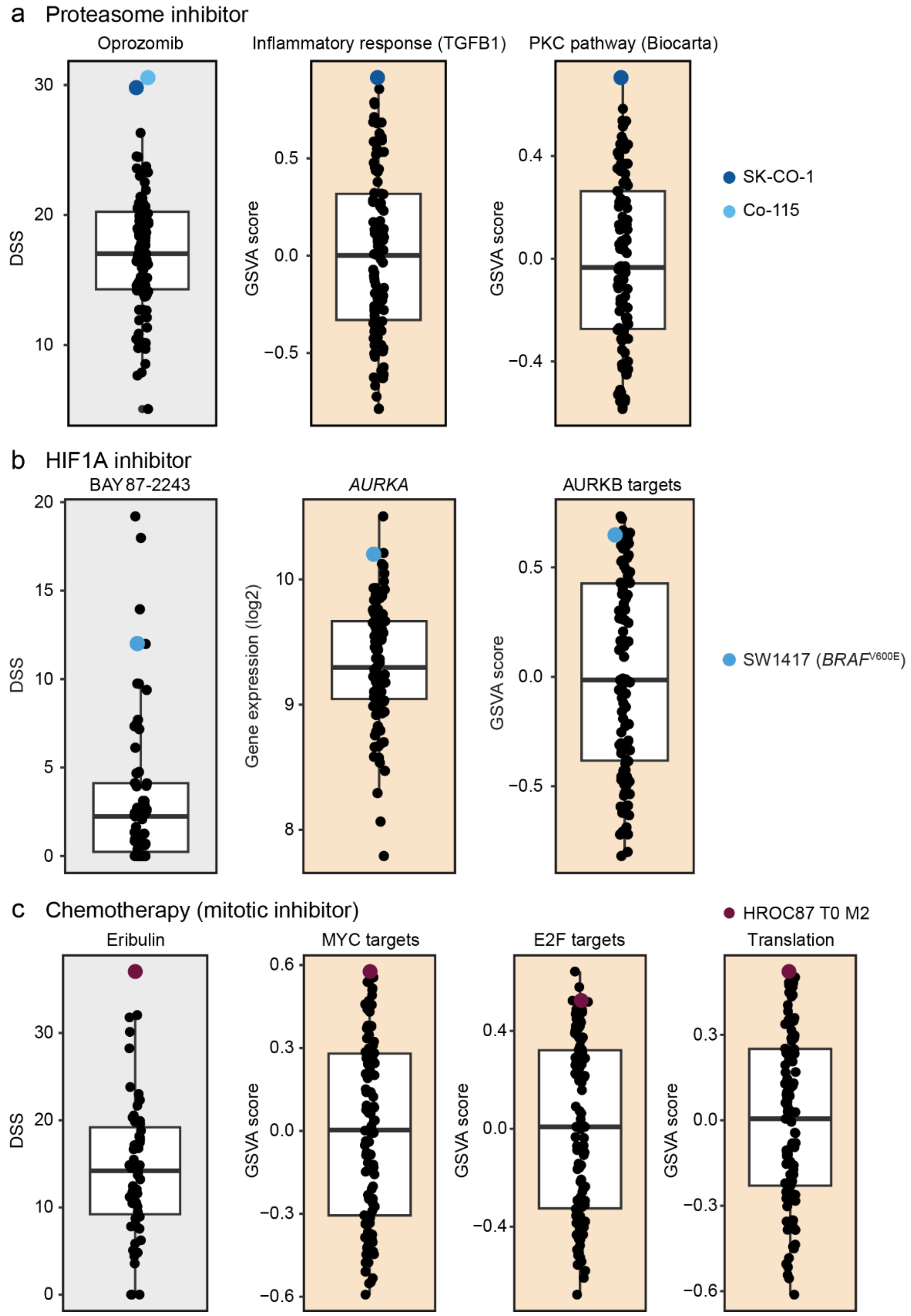

Fig. S16 Molecular underpinnings of exceptional sensitivity to a proteasome inhibitor, HIF1A inhibitor, and the chemotherapy eribulin

Box plot of DSS values of (**a**) the proteasome inhibitor oprozomib, (**b**) the HIF1A inhibitor BAY 87-2243, and (c) the chemotherapeutic agent eribulin (mitotic inhibitor) among cell lines (gray background). Cell lines with exceptional sensitivity are highlighted in color as indicated. Box plots with orange background show single-sample enrichment scores (GSVA scores) of selected gene sets or gene expression values of *AURKA*. The cell line Co-115 in panel a did not have a molecular association to the exceptional drug sensitivity.

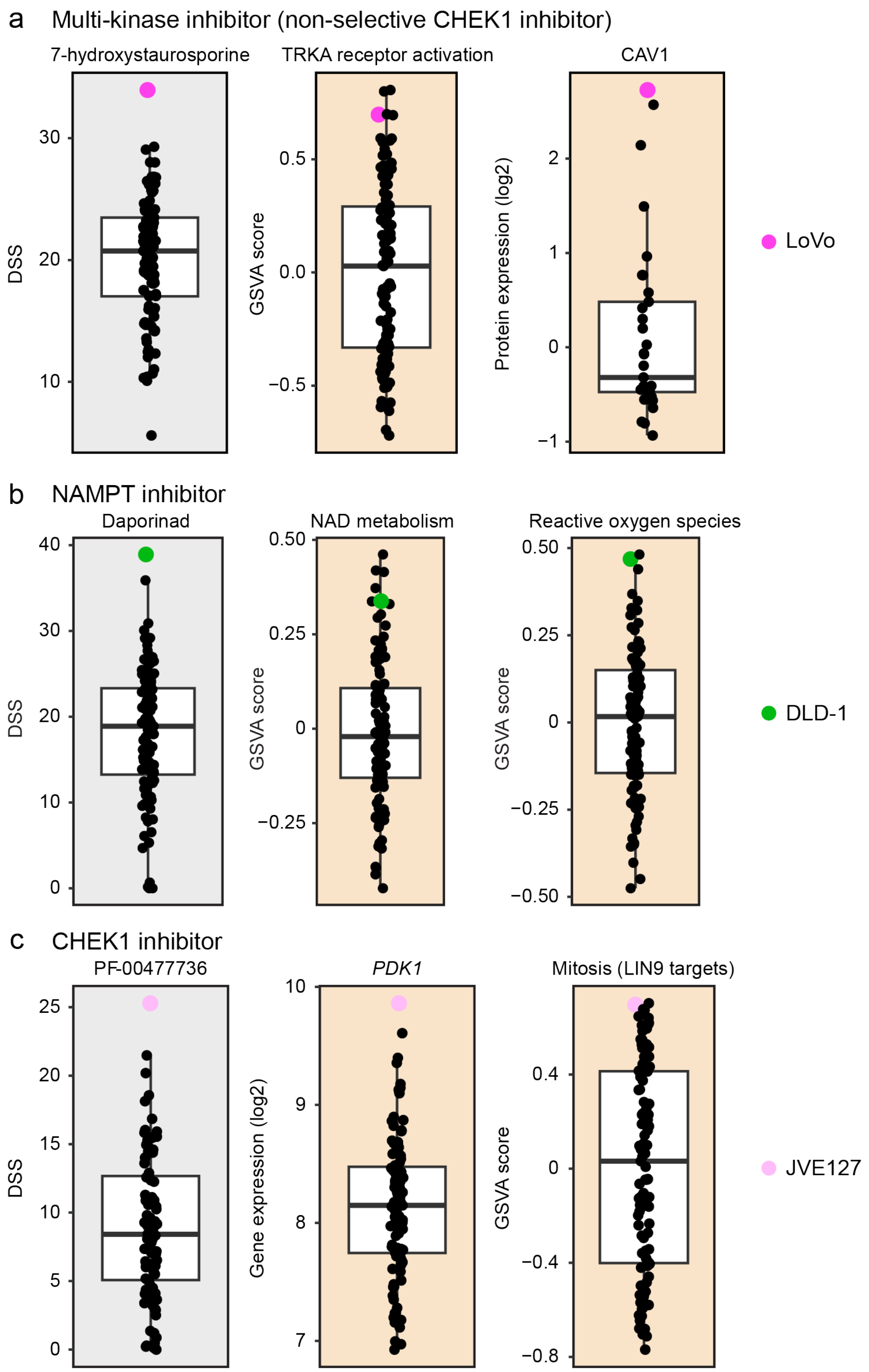

Fig. S17 Molecular underpinnings of exceptional sensitivity to a multi-kinase, NAMPT, and CHEK1 inhibitor

Box plot of DSS values of (**a**) the non-selective CHEK1 inhibitor 7-hydroxystaurosporine, (**b**) the NAMPT inhibitor daporinad, and (**c**) the CHEK1 inhibitor PF-00477736 among cell lines (gray background). Cell lines with exceptional sensitivity are highlighted in color as indicated. Box plots with orange background show single-sample enrichment scores (GSVA scores) of selected gene sets, protein expression of CAV1 (median centered), or gene expression values of *PDK1*.

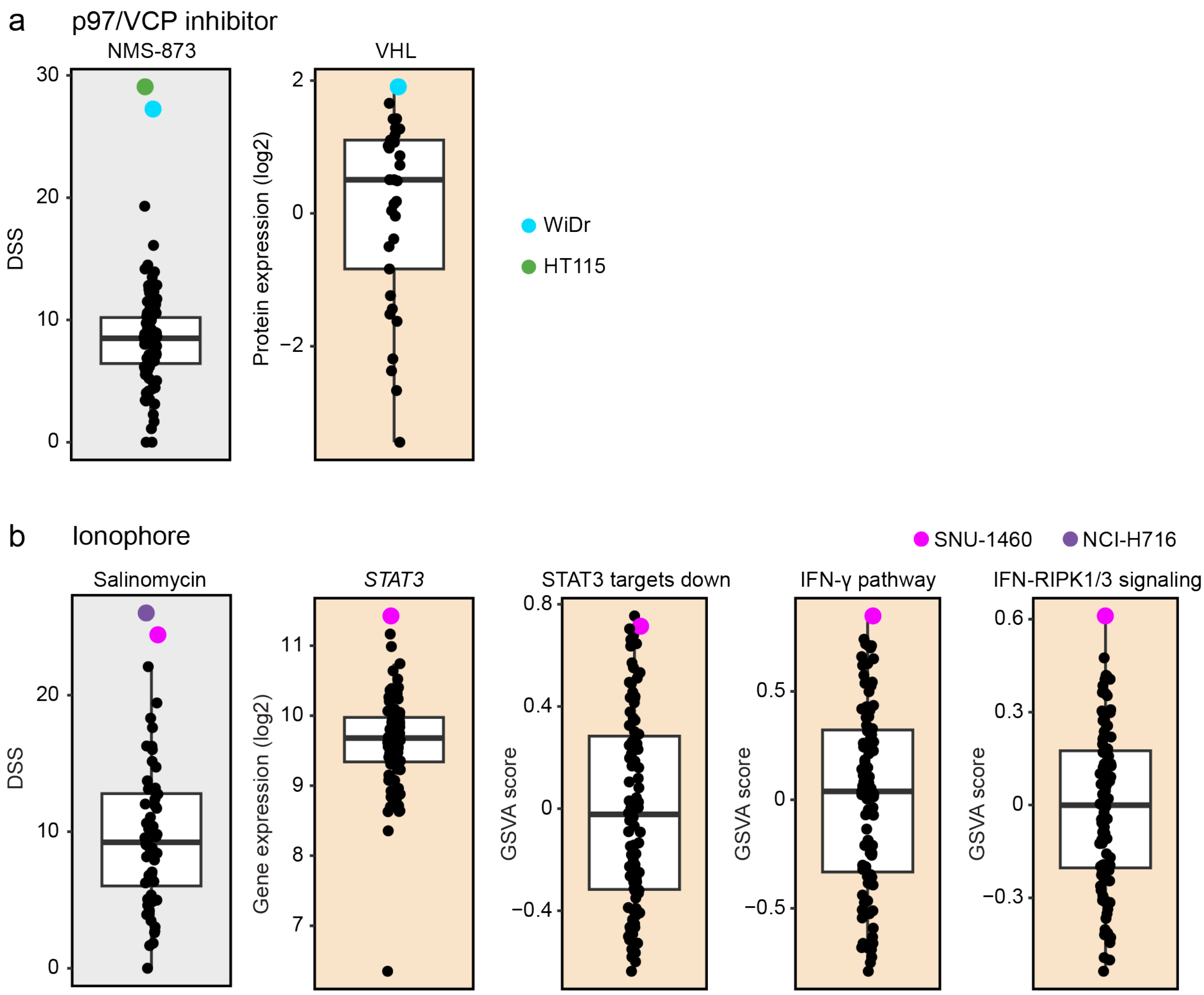

Fig. S18 Molecular underpinnings of exceptional sensitivity to a p97/VCP inhibitor and ionophore

Box plot of DSS values of (**a**) the p97/VCP inhibitor NMS-873 and (**b**) the ionophore salinomycin among cell lines (gray background). Cell lines with exceptional sensitivity are highlighted in color as indicated. Box plots with orange background show protein expression of VHL (median centered), gene expression values of *STAT3*, or single-sample enrichment scores (GSVA scores) of selected gene sets. The cell lines HT115 (panel a) and NCI-H716 (panel b) did not have a molecular association to the exceptional drug sensitivity.

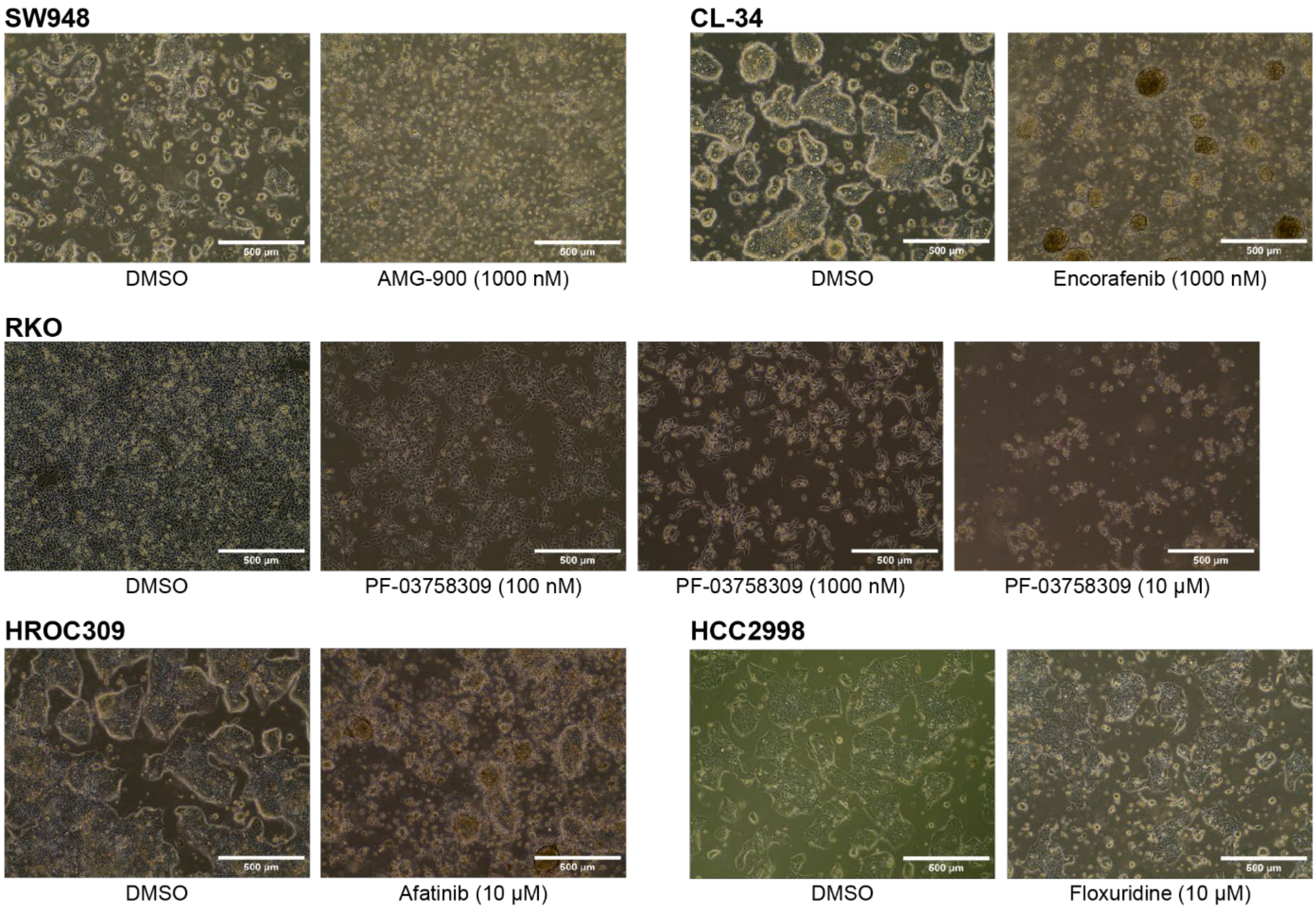

Fig. S19 Micrographs of cell cultures after drug treatment in validation experiments

Micrographs of 7 of the cell lines selected for validation experiments after 48 h treatment with DMSO (control) and the highest concentration(s) of the drug with exceptional activity. The scale bar is 500 µm.

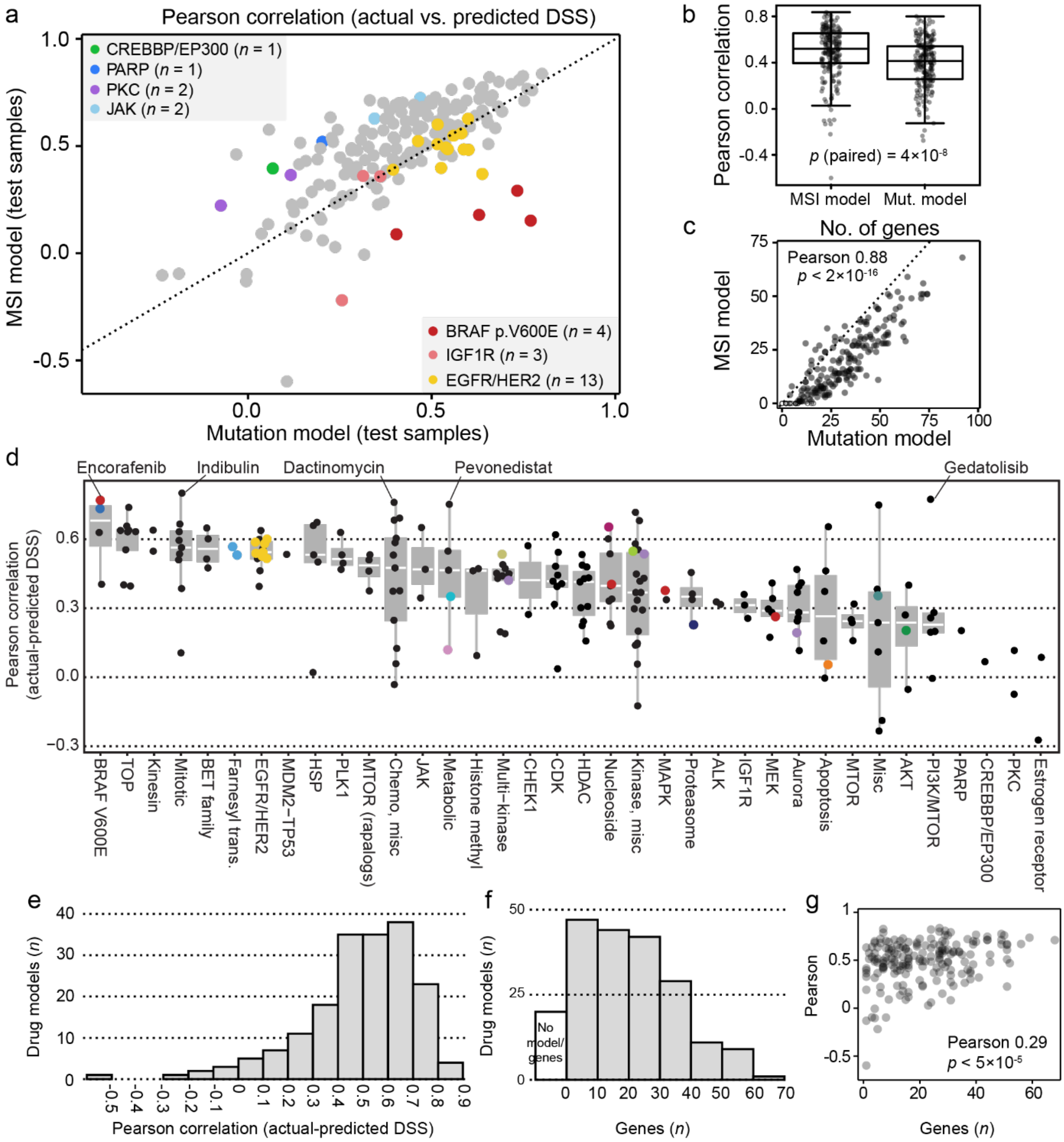

Fig. S20 Performance and feature selection for transcriptomic predictions models of drug sensitivity using MADMMplasso

(**a**) Scatter plot of Pearson correlations between actual and predicted DSS for each drug (*n* = 183; no model/genes for 20 drugs) among the test samples in models with MSI status or mutations as the modifying term. Drugs were grouped according to molecular target (Table S6), and the groups with the largest mean difference between the MSI and mutation models are highlighted in colors. (**b**) Box plot of Pearson correlations between actual and predicted DSS of each drug in the MSI and mutation models. P-value is from paired t-test. (**c**) Scatter plot of the number of genes selected for the prediction model of each drug with MSI status or mutations as the modifying term. (**d**) Box plot of Pearson correlations between actual and predicted DSS of each drug in the test samples with mutations as the modifying term. Drugs are grouped according to molecular target and ordered according to decreasing median correlation. The top 5 drugs are specified. Modeled drugs with

exceptional activity (*n* = 26) are highlighted in color. Histogram of (**e**) Pearson correlation coefficients between the actual and predicted DSS of each modeled drug in the test set of samples and (**f**) the number of genes (features) selected for each drug model with MSI status as modifying term. (**g**) Scatter plot of Pearson correlations between actual and predicted DSS versus the number of genes selected for each model with MSI status as modifying term.

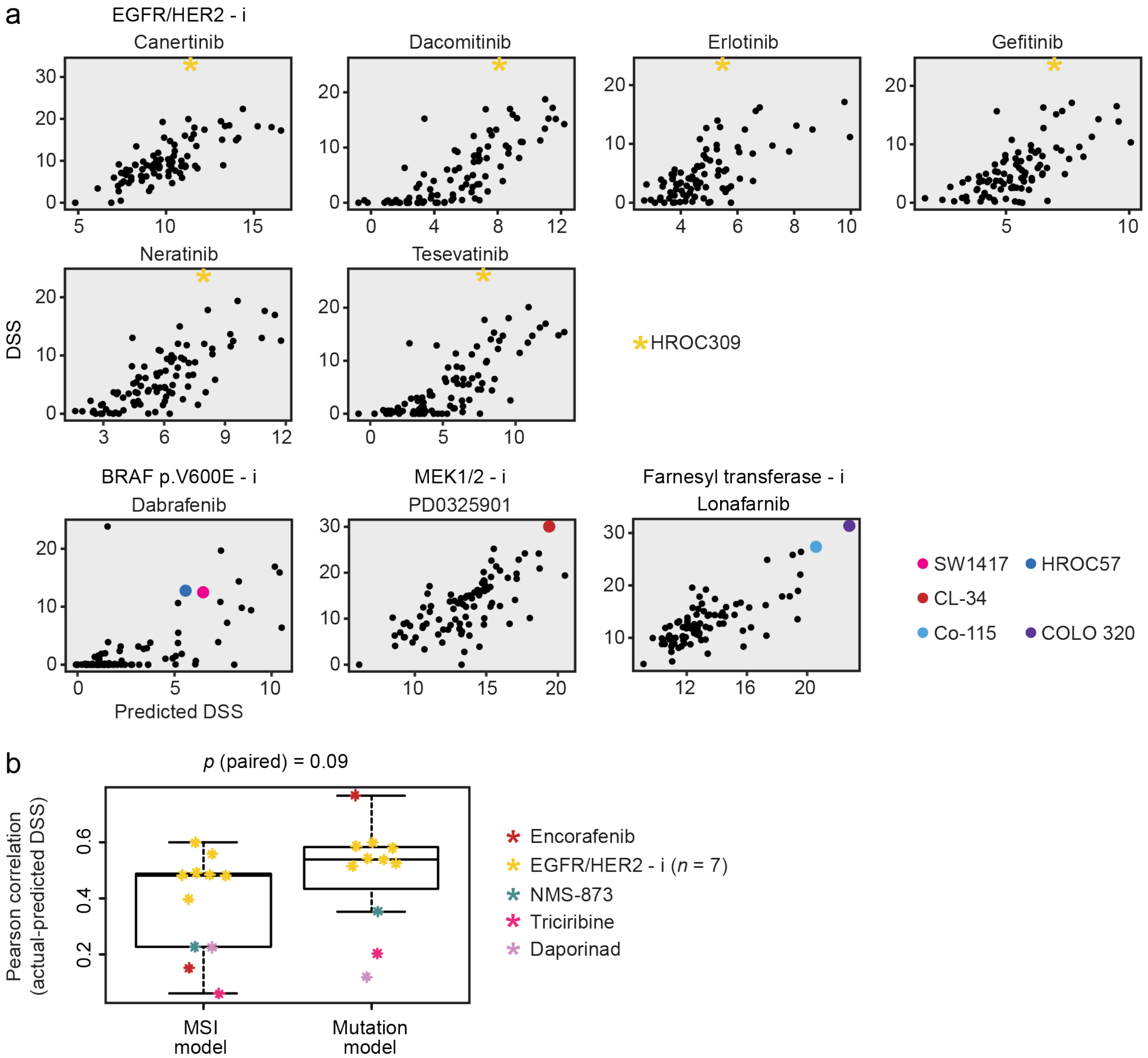

Fig. S21 Predicted and actual DSS of drugs with exceptional activity

(**a**) Scatter plots of actual (vertical axes) and predicted (horizontal axes) DSS of drugs with exceptional activity. Cell lines with exceptional sensitivity are indicated by colors, and exceptional sensitivities considered not to be predicted are marked as asterisks. This plot supplements main Fig. 6b and includes drugs with the same mechanism of action as the drugs included in the main figure. (**b**) Boxplot of performance of the MSI model versus mutation model for drugs with exceptional sensitivities that were not well predicted with the MSI model.

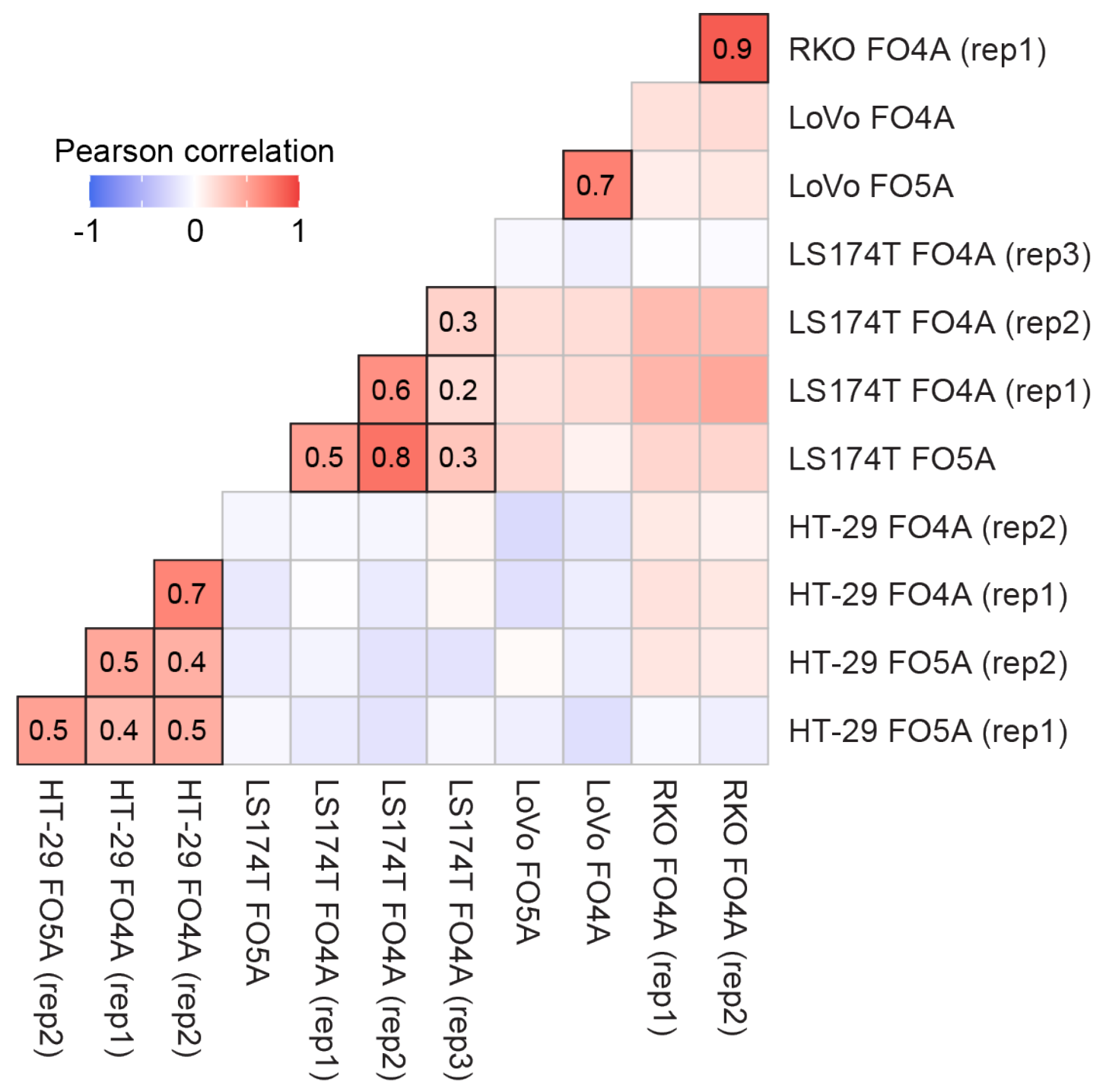

Fig. S22 Correlations of replicate drug screens

Heatmap of Pearson correlations of cell lines (*n* = 4) screened for both drug libraries (FO4A and FO5A) and/or in replicates over time (rep1-3). DSS values were centered to a mean of 0 and scaled to a standard deviation of 1 across all samples included in the screen, and correlations between samples were calculated among overlapping drugs in the two libraries (*n* = 369).

Supplementary Methods

Gene expression analyses

Transcriptomic profiling was performed with Affymetrix Human Transcriptome 2.0 arrays using 100 ng of total RNA as input and following the manufacturer's protocol (Thermo Fisher Scientific, Waltham, MA, USA). Raw intensity CEL-files were processed according to the robust multi-array average approach (Irizarry et al., Biostatistics 2003;4:249–64) using the function justRMA in the R package affy (v. 1.84.0) (Gautier et al., Bioinformatics 2004;20:307–15) and custom CDF files from Brainarray (hta20hsentrezgcdf_25.0.0) (Dai et al., Nucleic Acids Res 2005;33:e175). Gene annotations according to the GRCh38.p13 genome assembly were retrieved using the function getBM in the R package biomaRt (v. 2.58.0) (Durinck et al., Nat Protoc 2009;4:1184–91). Protein-coding genes annotated with unique HGNC symbols were retained (n = 18,739).

Transcriptomic similarity to CRC tissue was evaluated using the R package CancerCellNet (v. 0.2.0). A classifier was trained on tumor tissue samples from The Cancer Genome Atlas using the function broadClass_train with default settings, and cell lines were scored using the function broadClass_predict. Classification according to the consensus molecular subtypes (CMS) was performed using the R package CMScaller (v. 2.0.1) with default settings. Classification according to the intrinsic CMS (iCMS) was performed using the approach and gene template described in the original publication (Joanito et al., Nat Genet. 2022;54:963–75). Gene expression estimates were centered by the mean and scaled by the standard deviation across samples using the function ematAdjust, and samples were classified with nearest template prediction using the function ntp in CMScaller and a threshold for the false discovery rate according to the Benjamini-Hochberg method of 0.05.

External validation dataset

The Genomics of Drug Sensitivity in Cancer (GDSC2) dataset was downloaded from Cell Model Passports (https://cmp.cog.sanger.ac.uk/download/GDSC2_fitted_dose_response_27Oct23.xlsx) on March 31, 2026. Drug sensitivity estimates in the GDSC2 dataset (natural logarithm of the fitted IC50 value, LN_IC50) were compared with DSS values for overlapping cell lines (n = 34) and drugs (n = 130) in the in-house dataset. Drugs with frequent missing values in either dataset (in 12 or more samples, remaining drugs had less than 5 missing values) and/or low variation of activity in the in-house dataset (cross-sample 10-90th percentile range < 5) were excluded from drug-wise correlation analyses (n = 67).

Validation experiments with drug treatment and Western blotting

Cell lines (n = 7) selected based on exceptional drug sensitivities were seeded in 6-well plates and incubated at 37 °C with 5% CO_2 until reaching approximately 70% confluency. Treatment was performed by 48 h incubation with drugs manually supplied in fresh medium at five different concentrations (n = 7 drugs in the same concentrations as the automated high-throughput drug screen; Table S5) and with vehicle control (0.01% w/v DMSO). Cells were washed twice with Gibco^{TM} phosphate buffered saline (Thermo Fisher Scientific), lysed in 300 µL SDS sample buffer (10 mm Tris (pH 6.8), 15% w/v glycerol, 3% w/v SDS, 0.01% w/v bromophenol blue, and 5% v/v 2-mercaptoethanol) and collected in 1.5 ml tubes with a cell scraper. Samples were then sonicated at 37% amplitude for 25 seconds and proteins were denatured on a 95 °C heat block for 5 minutes.

Protein samples were separated on 10% pre-cast gels and transferred onto PVDF membranes using the Trans-Blot Turbo Transfer System (Bio-Rad Laboratories, Inc., Hercules, California, USA). Membranes were blocked for 1 h with 5% non-fat dry milk (Cell Signaling Technology, Inc., Danvers, Massachusetts, USA) in 0.1% TBS-Tween 20 (TBS-T; Thermo Fisher Scientific), incubated overnight with primary antibodies at 4 °C, washed 4 × 5 min with TBS-T, and incubated 1 h with the respective secondary antibodies at room temperature. Antibodies were diluted either in 5% non-fat dry milk in TBS-T for chemiluminescent detection or Intercept TBS Blocking Buffer (LI-COR Biosciences, Lincoln, Nebraska, USA) for fluorescent detection (Table S5). All blots were washed 3 × 5 min with TBS-T and stored in TBS before development. Chemiluminescent blots were developed using SuperSignal West Dura Extended Duration Substrate (Thermo Fisher Scientific) for 5 min. All blots were imaged with the ChemiDoc MP System (Bio-Rad), and band intensities were quantified using Image Lab software version 6.1.0 (Bio-Rad). The blots were run once per sample and experimental condition.

Transcriptomic prediction of drug sensitivity

Transcriptomic prediction models of drug sensitivity were constructed using MADMMplasso (v. 1.0.0) in R (v. 4.1.3). This is a method for regularized tree-guided multi-response regression with main and interaction effects. Modeling was performed with the most variable gene expression features (explaining 50% of the total variation across cell lines) on the common set of drugs between the two libraries after filtering based on drug activity (*n* = 203; Table S2). Information of drug similarity based on complete-linkage hierarchical clustering of the Euclidean distances of DSS values was included as input to allow joint modeling of correlated drugs. Modeling was performed twice, using either MSI status or gene mutations (*BRAF* p.V600E, *RAS* hotspots [*KRAS*/*NRAS* p.G12, p.G13, p.Q61, p.K117, p.A146], *PIK3CA*, and *TP53*) for potential interactions (labelled the "MSI model" and "mutation model", respectively). MSI status or each mutation were included as binary modifying variables in the respective model. Cell lines with several missing DSS values due to low strictly standardized mean differences of drug plates were excluded (*n* = 12; Table S1). The data were split into training (70%) and test (30%) sets in ten repetitions with different random splits. The best model for each split was chosen using 5-fold cross-validation on the training data. Final models were obtained based on summaries of all 10 repetitions, and features or variables that appeared more than two times in all 10 repetitions were selected to ensure stability. Performance was evaluated in the test samples based on Pearson correlations between the actual and predicted DSS for each drug, using results from the first random repetition. Potential to predict exceptional sensitivities was evaluated for the cell-line drug pairs included in the MADMMplasso model based on Pearson correlations of actual and predicted DSS among all samples (training and test set combined).
